# A role for nucleosome remodellers during resection of deprotected telomeres in yeast

**DOI:** 10.1371/journal.pone.0352656

**Published:** 2026-07-10

**Authors:** Anja M. Deiser, Caitlyn Agar, Juan de Dios Barba Tena, Boris Pfander

**Affiliations:** Department of Chemistry and Chemical Biology, Cell Biology, Dortmund Life Science Center (DOLCE), TU Dortmund University , Dortmund, Germany; Tulane University Health Sciences Center: Tulane University School of Medicine, UNITED STATES OF AMERICA

## Abstract

DNA double-strand break (DSB) repair pathway choice is strongly influenced by DNA end resection, a process in which the 5′ DNA strand is degraded to generate 3′ single-stranded DNA required for homologous recombination. Although the enzymatic mechanisms of resection have been well defined, its regulation by the dynamic chromatin environment surrounding the DNA break remains less clear. Here, we used the budding yeast *cdc13-1* system to analyse DNA end resection after telomere deprotection. Inactivation of Cdc13, a component of the CST (Cdc13–Stn1–Ten1) telomere-capping complex, exposes telomeric DNA ends and triggers a DNA damage response. Using this system, we examined the contribution of long-range resection nucleases and chromatin regulators. Analysis of long-range resection nucleases revealed that the Dna2 nuclease contributes to telomeric processing, particularly in the absence of the exonuclease Exo1. Genetic removal of chromatin regulatory factors showed that H2A.Z, a histone H2A variant incorporated by the SWR1 complex, did not significantly affect resection, whereas depletion of the major nucleosome eviction complexes RSC and SWI/SNF impaired resection after telomere deprotection. Together, these results indicate that nucleosome eviction allows for efficient resection at deprotected telomeres and the analogies to long-range resection at DSBs illustrate the utility of the *cdc13-1* system for studying long-range resection in broader context.

## Introduction

DNA double-strand breaks (DSBs) represent a severe form of DNA damage and are a major source of genome instability. However, DSBs are also actively induced during meiosis and lymphocyte development, as well as in gene-editing procedures [1–3]. Eukaryotic cells employ several pathways to repair DSBs, each with different attributes including mutagenic potential. Consequently, the choice of the DSB repair pathway is a key determinant of genome stability. A central factor influencing this choice is DNA end resection. Mechanistically, resection refers to the nucleolytic processing of DSB ends [1,4]. involving degradation of the 5′ end and generation of a 3′ single-stranded DNA (ssDNA) overhang. Such ssDNA overhangs are required for repair by homologous recombination (HR). Resection can also expose microhomologies that promote repair through microhomology-mediated end joining (MMEJ) [5], an intrinsically mutagenic process. In contrast, resection counteracts DSB repair by non-homologous end-joining (NHEJ), as it removes the substrate for end-ligation [6].

In eukaryotes, DSB resection is carried out by three evolutionary conserved resection nucleases and proceeds via a two-step mechanism involving initiation (short-range resection) and elongation (long-range resection) [7,8]. Short-range resection is mediated by the endo-/exonuclease Mre11 functioning within the Mre11 complex (Mre11-Rad50-Xrs2 (MRX) in budding yeast and MRE11-RAD50-NBS1 (MRN) in humans) together with its activator Sae2 in budding yeast or CTIP in human cells [1,4]. Short-range resection includes endonucleolytic cleavage by the Mre11 complex followed by 3′–5′ exonucleolytic processing towards the break site. While short-range resection is not strictly required for “clean” DSBs—for instance those generated by restriction enzymes—it plays a crucial role in removing protein blocks from DNA ends [9–11]. Generally, short-range resection generates an entry point for long-range processing which extends the 3′ overhangs up to several thousand nucleotides [1,4].

Long-range resection can be catalysed by either the Exo1 exonuclease (EXO1 in humans) or the multifunctional helicase/endonuclease Dna2 (DNA2 in humans), which acts together with the STR complex (Sgs1-Top3-Rmi1 in budding yeast or BLM-TOP3A-RMI1/2 in humans) [1,4,7,8]. Resection activity is tightly regulated throughout the cell cycle and becomes upregulated during S-phase once a sister chromatid becomes available as a template for HR-dependent repair [12]. At least two cell-cycle kinases—cyclin-dependent kinase (CDK) and Dbf4-dependent kinase (DDK)—directly contribute to this upregulation by phosphorylating resection nucleases and associated factors [12–17]. Biochemical reconstitution of DSB resection using budding yeast and human proteins has provided detailed insights into its molecular mechanisms and regulation [18,19].

In contrast to these advances, our understanding of how resection proceeds on chromatin remains limited. Nucleosomes play multiple roles during the DNA damage response: They serve as platforms for signalling events triggered by DSBs and facilitate recruitment of repair proteins [20]. Notably, experiments in budding yeast indicate that nucleosomes are evicted concomitant with resection and that such eviction is in turn required for resection [21]. These findings align with *in vitro* evidence suggesting that nucleosomes hinder nuclease access during resection reactions themselves [22]. Accordingly, nucleosome remodellers – ATP-driven molecular machines that often consist of multiple proteins and catalyse sliding, eviction and editing of nucleosomes – emerge as key facilitators and regulators of resection [23,24]. However, their study is complicated due to the functional overlap of different remodeller complexes and their contribution to multiple aspects of genome biology. Moreover, certain remodellers such as yeast Fun30 are hypothesised to act on specific nucleosome subpopulations and nucleosome-associated proteins [15,16,24–28].

Much of our current knowledge about DSB repair stems from experiments using site-specific induction of DSBs using HO-endonuclease [2], restriction endonucleases [21,29,30] or more recently CRISPR-Cas9 [31]. Site-specific induction enables investigation of repair dynamics at distinct genomic loci and thus allows assessment of how local chromatin environments influence DSB resection and other repair outcomes. However, this approach relies critically on efficient delivery systems. The interpretation of experimental results becomes challenging when experimental conditions lead to poor DSB induction.

In the budding yeast model, deprotected telomeres offer another possibility to study DNA end resection. Specifically, thermal inactivation of the temperature-sensitive *cdc13-1* allele triggers extensive telomeric resection at roughly 10–20% of telomeres [32,33]. The formation of ssDNA can be quantified using QAOS (Quantitative Amplification Of Single-stranded DNA) [33,34]. In healthy cells, Cdc13 is part of the CST-complex (Cdc13-Stn1-Ten1), which specifically binds to the single-stranded 3’ overhang at telomeres, and has been shown to also bind double-stranded/single-stranded DNA junctions *in vitro* [35,36]. Thereby CST caps the telomere and counteracts resection together with Rap1 and the Ku-complex [37–39]. Upon decapping, exposure of the telomeric ssDNA leads to recognition of the DNA repair machinery. So far, the differences of telomeric versus intrachromosomal resection are not fully understood. In particular, budding yeast telomeres and adjacent subtelomeric regions exhibit a distinct chromatin organisation [40], which may affect resection. Budding yeast telomeres appear largely devoid of nucleosomes but are bound by Rap1—a protein critical for recruiting Sir2–4 and Rif1–2 effectors—as well as Reb1/Tbf1 [38,41–44]. Next to the sub-telomeric domains, nucleosomes are well positioned and occur together with sequence-specific binding proteins such as ORC and Abf1 [40]. Moreover, telomeres can be transcribed by RNA polymerase II into a long non-coding RNA TERRA, which has been associated with different telomere regulatory functions, in particular involved in telomere capping and chromatin regulation [45–47]. Given this specialised architecture at chromosome ends, it remains unclear whether resection after telomere deprotection undergoes processing similar to intrachromosomal DSBs or requires additional factors.

In this study, we used the budding yeast *cdc13-1* system to investigate resection after telomere deprotection. An initial focus on long-range resection nucleases revealed that Dna2 contributes to long-range resection after telomere deprotection and gains importance in the absence of Exo1. With regard to chromatin dynamics, we could not detect a significant effect on resection by nucleosome editing or H2A.Z incorporation. In contrast, depletion of the major nucleosome evictors RSC and SWI/SNF abrogated resection after telomere deprotection, consistent with our previous data using enzyme-induced DSBs [21]. In all, these similarities highlight the potential of the *cdc13-1* system to reveal regulators not only for resection of deprotected telomeres, but of DSB repair in general.

## Results

### Dna2 contributes to long‑range resection after telomere deprotection

In budding yeast, two long-range resection pathways mediated by Exo1 and Dna2 with the STR complex, respectively, were found to contribute to resection of enzyme-induced DSBs [7,8]. Upon telomere deprotection by *cdc13-1* inactivation, Exo1 appears to be majorly responsible for long-range resection of deprotected telomeres [34]. Currently, a role for Dna2 has not been investigated due to a lack of genetic tools. However, the helicase Sgs1 has been shown to contribute to resection after telomere deprotection in an alternative pathway to Exo1 and the effect became apparent especially in the absence of Rad9 [34].

**Fig 1:**
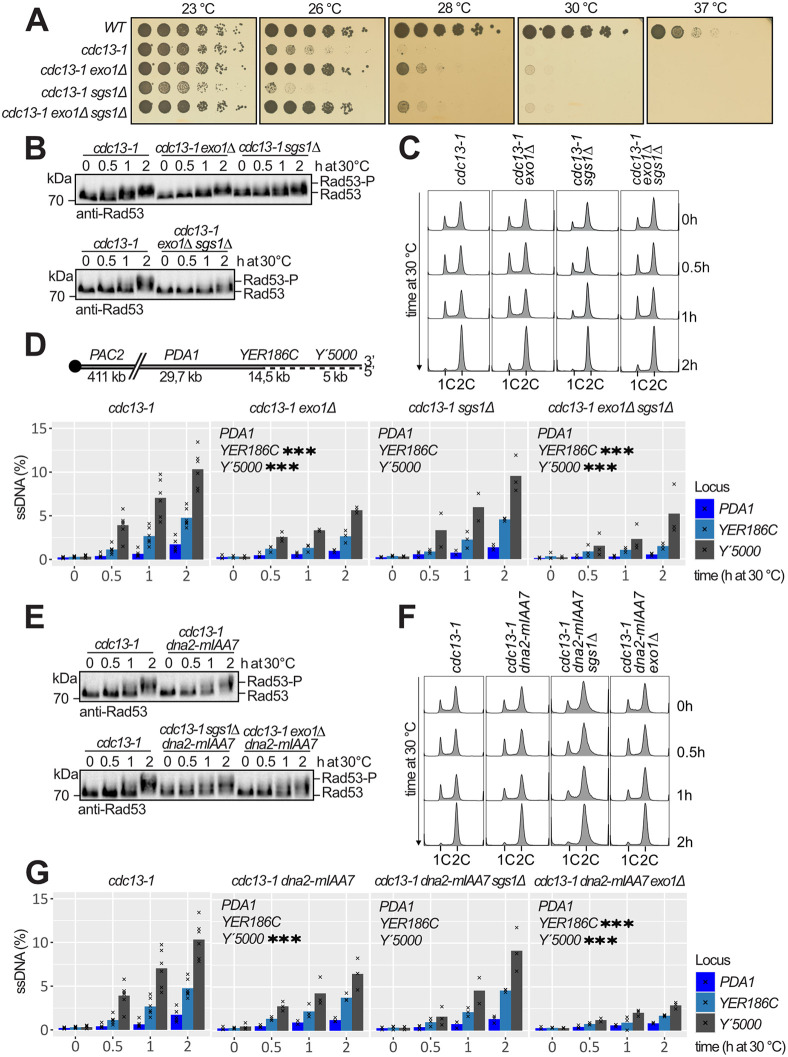
Dna2 is involved in resection of deprotected telomeres. **(A)** Growth of *cdc13-1* mutant strains at different temperatures: Five-fold serial dilutions of *WT, cdc13-1, cdc13-1 exo1∆, cdc13-1 sgs1∆ and cdc13-1 exo1∆ sgs1∆* were spotted on YPD plates and incubated for 48 h at indicated temperatures. Data is representative of n = 2–4 biological replicates. **(B)** Rad53 activation after telomere deprotection in *cdc13-1* mutant strains: Western Blots detecting phosphorylated forms of Rad53 visualised by gel shift show increasing levels of phosphorylated Rad53 after temperature shift to 30 °C of *cdc13-1, cdc13-1 exo1∆, cdc13-1 sgs1∆ and cdc13-1 exo1∆ sgs1∆*. Data is representative of n = 2–4 biological replicates. **(C)** Cell cycle arrest after telomere deprotection in *cdc13-1* mutant strains: Mutant strains were grown at 23°C, temperature shifted to 30 °C and samples taken at indicated timepoints. Total DNA content was measured by flow cytometry after SYTOX green staining. Data is representative of n = 2–4 biological replicates. **(D)** Resection of deprotected telomeres of Chr V was measured by quantitative amplification of ssDNA (QAOS): Yeast cultures of c*dc13-1, cdc13-1 exo1∆, cdc13-1 sgs1∆ and cdc13-1 exo1∆ sgs1∆* were shifted to 30 °C and harvested at indicated timepoints. Accumulation of ssDNA was measured at *Y´5000*, *YER186C* and *PDA1* loci. Data is representative of n = 3−6 biological replicates. The significance of differences in ssDNA accumulation compared to the *cdc13-1* control strain was calculated using a linear mixed-effects model. **(E)** Temperature shift of *cdc13-1* mutants induces Rad53 activation: Western Blots detecting phosphorylated forms of Rad53 as in (B) but with *cdc13-1, cdc13-1 dna2-mIAA7, cdc13-1 sgs1∆ dna2-mIAA7* and *cdc13-1 exo1∆ dna2-mIAA7.* Data is representative of n = 2–3 biological replicates. **(F)** Cell cycle arrest of *cdc13-1* mutants after temperature shift: Flow cytometry as in (C) but with *cdc13-1, cdc13-1 dna2-mIAA7, cdc13-1 sgs1∆ dna2-mIAA7* and *cdc13-1 exo1∆ dna2-mIAA7.* Data is representative of n = 2 biological replicates. **(G)** Resection of deprotected telomeres in mutant strains after auxin treatment: Measured by QAOS as in (D) but with *cdc13-1, cdc13-1 dna2-mIAA7, cdc13-1 sgs1∆ dna2-mIAA7* and *cdc13-1 exo1∆ dna2-mIAA7.* Data is representative of n = 3−6 biological replicates. 6 biological replicates of the *cdc13-1* control strain have been pooled and are identical as in Fig 1D.

We established an experimental pipeline, in which we investigated *cdc13-1*-dependent telomere deprotection upon temperature shift. Consistent with previous results [32–34], we observed an escalating, temperature-dependent growth defect of a *cdc13-1* mutant yeast strain from 26 °C to 37 °C (Fig 1A). An acute temperature shift from 23 °C to 30 °C led to phosphorylation of the DNA damage checkpoint kinase Rad53 (Figs 1B and S1A, S1B Figs) and G2/M cell cycle arrest (Fig 1C). Resection after telomere deprotection was measured by quantitative amplification of single‑stranded DNA (QAOS) at chromosome V, specifically at a sub-telomeric locus (*Y’5000*), as well as at two genomic loci (*YER186C* and *PDA1*). The three tested loci are located at distances 5 kB (*Y’5000*), 14.5 kB (*YER186C*) and 29.7 kB (*PDA1*) from the right end of chromosome V (Fig 1D). We normalised to an internal locus (*PAC2*) and used ssDNA/dsDNA dilution series to obtain relative amounts of resected ssDNA (S1C Fig). By QAOS, we found that after temperature-shift to 30 °C, up to 10% −15% of the sub-telomeric *Y’5000* locus became single-stranded, while we did not observe increased ssDNA at 23 °C (Fig 1D and S1D Fig). The *cdc13-1 exo1*Δ strain accumulated significantly less ssDNA, whilst the resection defect in the *cdc13-1 sgs1*Δ strain was minor and not significant (Fig 1D and S1E Fig). Combining mutants in the *cdc13-1 sgs1*Δ *exo1*Δ showed almost no ssDNA generation at *YER168C* and *PDA1* indicating that Sgs1 is involved in resection in the absence of Exo1 (Fig 1D and S1E Fig).

As Dna2 works together with Sgs1 at DSBs, we aimed to assess whether Dna2 acts in the resection of deprotected telomeres, as well. Because Dna2 is essential, we made use of conditional depletion. Specifically, we used a miniIAA7 degron cassette, which we added to the C-terminus of Dna2, constitutively overexpressed *OsTIR1* and added synthetic auxin (Indole-3-acetic acid, IAA) [48,49]. Addition of IAA led to efficient degradation of Dna2 within 1h (S1F Fig) and loss of viability of *dna2-mIAA7* strains (S1G Fig), consistent with the essential nature of *DNA2*. We combined Dna2 degradation with telomere deprotection in a *cdc13-1 dna2-mIAA7* double mutant strain and depleted Dna2 prior to shifting temperature and inducing telomere deprotection. When telomere deprotection was induced after Dna2 degradation, we observed cell cycle-arrest, but slightly reduced Rad53 phosphorylation, when compared to *cdc13-1* cells (Figs 1E, 1F), consistent with a role of Dna2 in checkpoint activation [50]. In the same experiments, we measured resection after telomere deprotection by QAOS and found reduced resection in *cdc13-1 dna2-mIAA7* cells (Fig 1G and S1H Fig). Compared to the *cdc13-1* control, resection was also slightly reduced in *cdc13-1 dna2-mIAA7 sgs1*Δ strains, but this effect was not significant (Fig 1G and S1H Fig). We tested whether Dna2 has a more pronounced contribution in the absence of Exo1. We generated a *cdc13-1 exo1*Δ *dna2-mIAA7* mutant and measured resection after telomere deprotection by QAOS and observed a strong decrease in the spreading of resection (Fig 1G and S1H Fig). We compared resection also to *cdc13-1 dna2-mIAA7* and *cdc13-1 exo1*Δ strains and found that resection was significantly increased in the *cdc13-1 exo1*Δ *dna2-mIAA7* strain (S1I Fig). We therefore conclude that Dna2 acts as a long-range resection nuclease at deprotected telomeres. Similar to the established mechanism of resection of DSBs in the genome, also at telomeres Exo1 and Dna2 appear to work in separate resection pathways, suggesting that similar long-range resection mechanisms operate at deprotected telomeres as at DSBs at other genomic locations [7,8].

### H2A.Z does not influence resection of deprotected telomeres

A key feature of dynamic chromatin is nucleosome editing, i.e., the exchange of histone subunits [51]. Budding yeast contains a single H2A variant called H2A.Z (encoded by the *HTZ1* gene). Consequently, H2A/H2A.Z-exchange is the predominant nucleosome editing mechanism in yeast. H2A.Z incorporation into chromatin is catalysed by the SWR1 nucleosome remodelling complex, while H2A.Z removal is thought to be catalysed by the INO80 complex [52–57]. Notably, previous work has demonstrated that the INO80 complex has a prominent role in the regulation of HR, but mainly at steps after DNA end resection, such as formation of the Rad51 filament [21,58–60]. In contrast, *in vitro* reconstitution experiments have suggested that H2A.Z incorporation into damaged chromatin could facilitate resection, consistent with H2A.Z-containing nucleosomes being less stable and posing less of a barrier for resection nucleases [22]. We studied resection of *cdc13-1* deprotected telomeres to investigate the influence of nucleosome editing on resection after telomere deprotection in three different genetic backgrounds: The *cdc13-1 arp8*Δ strain contains a dysfunctional INO80 complex due to the absence of the A-module [61,62]. Furthermore, the *cdc13-1 swr1*Δ *htz1*Δ strain lacks both the H2A.Z histone variant and the SWR1 complex responsible for its deposition. Using this double mutant avoids secondary phenotypes caused by persistent SWR1 complex activity causing additional defects in the absence of H2A.Z [63,64]. A third strain, *cdc13-1 arp8*Δ *swr1*Δ *htz1*Δ is deficient in both H2A.Z incorporating and removing activities, respectively.

**Fig 2:**
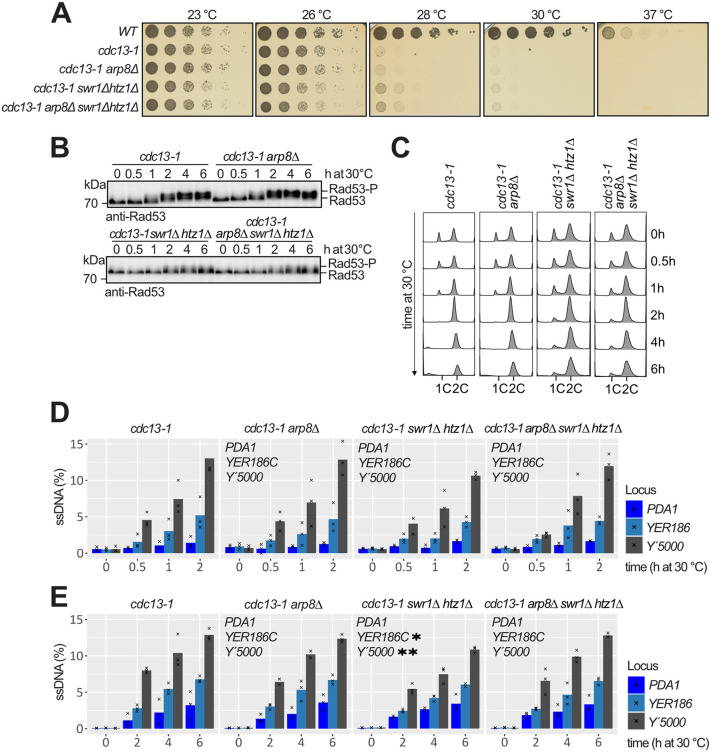
Nucleosome editing does not affect resection of deprotected telomeres. **(A)** Growth of *cdc13-1* mutants at different temperatures: Five-fold serial dilutions of *WT, cdc13-1, cdc13-1 arp8∆, cdc13-1 swr1∆ htz1∆* and *cdc13-1 arp8∆ swr1∆ htz1∆* were spotted on YPD and incubated for 48 h at indicated temperatures. Data is representative of n = 2 biological replicates. **(B)** Rad53 activation after telomere deprotection in *cdc13-1* mutants: Western Blots detecting phosphorylated forms of Rad53 visible by gel shift show increasing levels of phosphorylated Rad53 after temperature shift to 30 °C of *cdc13-1, cdc13-1 arp8∆, cdc13-1 swr1∆ htz1∆* and *cdc13-1 arp8∆ swr1∆ htz1∆*. Data is representative of n = 2–3 biological replicates. **(C)** Cell cycle arrest of *cdc13-1* mutants after telomere deprotection: Mutant strains were grown at 23 °C, temperature shifted to 30 °C and samples taken at indicated timepoints. Total DNA content was measured by flow cytometry after SYTOX green staining. Data is representative of n = 1–3 biological replicates. **(D)** Resection of deprotected telomeres of Chr V was measured by quantitative amplification of ssDNA (QAOS): Yeast cultures of *cdc13-1, cdc13-1 arp8∆, cdc13-1 swr1∆ htz1∆* and *cdc13-1 arp8∆ swr1∆ htz1∆* were shifted to 30 °C and harvested at indicated timepoints. Accumulation of ssDNA was measured at *Y´5000*, *YER186C* and *PDA1* loci. Data is representative of n = 3 biological replicates. The significance of resection defects in the mutant strains compared to the *cdc13-1* control was calculated using a linear mixed-effects model. A single biological replicate of the *cdc13-1* control strain is the same as in Fig 1D. **(E)** Resection of deprotected telomeres in chromatin remodeller mutants: Measured by QAOS as in (D) but for 0, 2, 4 and 6 h. Data is representative of n = 3 biological replicates.

None of the tested mutants suppressed the *cdc13-1* temperature sensitivity (Fig 2A). After inducing acute telomere deprotection by temperature shift of *cdc13-1* mutant strains, we observed reduced Rad53 phosphorylation and cell cycle arrest in all *cdc13-1* mutants lacking *SWR1* and *HTZ1* (Figs 2B, 2C). These data could be consistent either with reduced resection or deficient checkpoint signalling, respectively. Notably, when we measured resection of deprotected telomeres by QAOS, we observed similar resection in *cdc13-1*, *cdc13-1 arp8*Δ, *cdc13-1 swr1*Δ *htz1*Δ and *cdc13-1 arp8*Δ *swr1*Δ *htz1*Δ cells (Fig 2D and S2A Fig). To ensure we could use our experimental set-up to measure resection at the *PDA1* locus located 29.7 kb away from the deprotected telomere, we prolonged the time course after temperature shift to 2, 4 and 6 h (Fig 2E, see blue bars for *PDA1 locus*). Also in these experiments, resection was not consistently changed in the absence of H2A.Z, Swr1 or Arp8 (Fig 2E and S2B Fig). These results therefore indicated that deprotected telomeres are resected independently of nucleosome editing. The reduced Rad53 phosphorylation likely reflects an involvement of H2A.Z and the SWR1 complex in DNA damage checkpoint signalling without compromising DNA end resection, consistent with previous work [53].

### H2A.Z influences restriction enzyme-induced DSB induction

**Fig 3:**
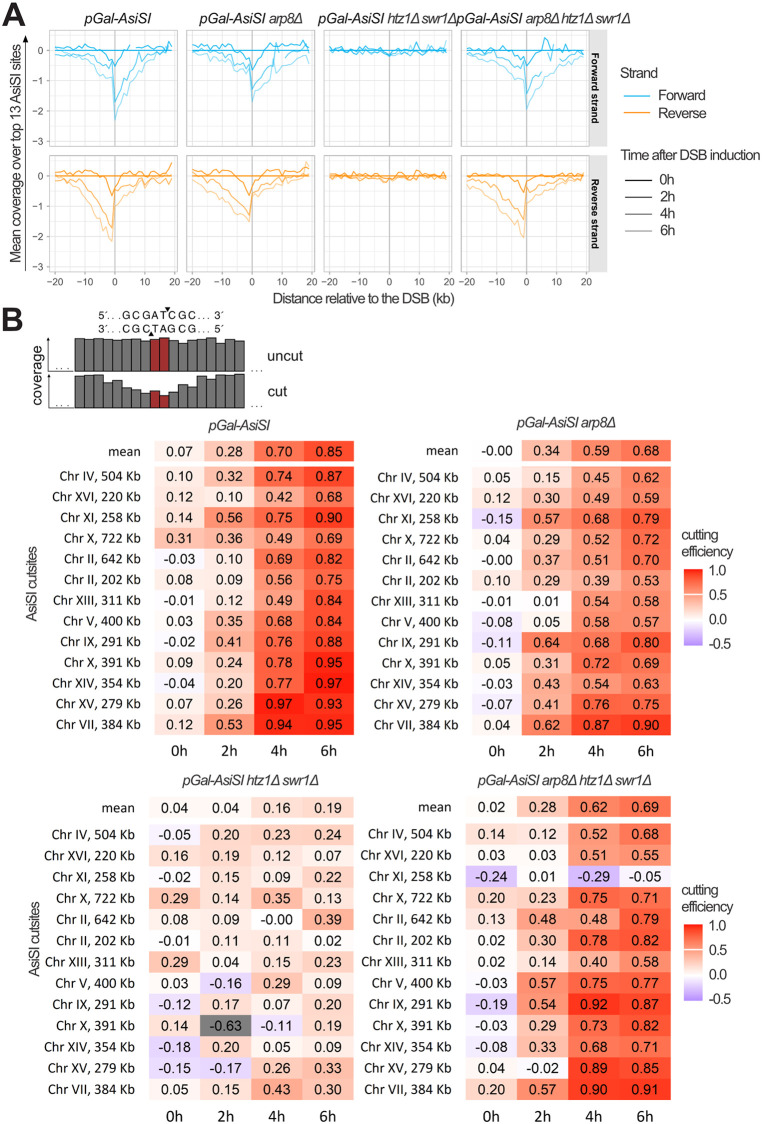
H2A.Z is required for AsiSI-dependent DSB induction. **(A)** Resection after restriction enzyme induced DSB induction was measured by strand-specific sequencing in *pGal-AsiSI, pGal-AsiSI arp8∆, pGal-AsiSI swr1∆ htz1∆* and *pGal-AsiSI arp8∆ swr1∆ htz1∆* strains. Samples are taken after induction of AsiSI at the indicated timepoints. The mean of DNA coverage over the top 13 resected AsiSI DSB sites is plotted with a window of 40 kb around the break sites. Data is representative of n = 2 biological replicates. **(B)** H2A.Z deficiency results in reduced AsiSI cutting: In parallel to the strand-specific sequencing experiment as described in (A), samples for Nanopore Sequencing were collected for *pGal-AsiSI, pGal-AsiSI arp8∆, pGal-AsiSI swr1∆ htz1∆* and *pGal-AsiSI arp8∆ swr1∆ htz1* strains. Cutting efficiencies were determined based on the coverage of the 2 bp AsiSI cutsite. Data is representative of n = 2 biological replicates.

We wanted to expand on these results from telomeric chromatin and test the influence of H2A.Z and the SWR1 or INO80 complexes on DSB resection more broadly. Therefore, we employed a system to simultaneously induce several DSBs at genomic locations using the AsiSI restriction enzyme expressed from the *pGAL1-10* promoter [21]. Previously, we showed that DSBs at 13 genomic locations can be induced efficiently (>50% cutting after 6 h) [21]. Notably, inefficient DSB-induction at other AsiSI sites correlated with nucleosome occupancy, consistent with the idea of DNA protection by nucleosomes [21]. We investigated resection at these 13 DSB sites 0, 2, 4 and 6 h after AsiSI-induction by strand-specific sequencing in *WT*, *arp8*Δ, *swr1*Δ *htz1*Δ and *arp8*Δ *swr1*Δ *htz1*Δ mutant strains, that have a defect in INO80 complex function, lack H2A.Z or combine both deficiencies (Fig 3A). We also evaluated DSB-induction (“cutting”) from the same samples using Nanopore Sequencing, in which we measure reduced coverage of the two nucleotide AT-overhang upon DSB induction (Fig 3B). Notably, we observed similar DSB induction in *WT*, *arp8*Δ and *arp8*Δ *swr1*Δ *htz1*Δ mutant strains (Fig 3B). Moreover, resection was comparable among *WT*, *arp8*Δ and *arp8*Δ *swr1*Δ *htz1*Δ mutant strains (Fig 3A) indicating that the INO80 complex does not have a major effect on DSB induction or resection. In contrast, we observed strongly reduced DSB-induction in *swr1*Δ *htz1*Δ cells (Fig 3B). Consistently, we also observed highly reduced DSB resection in the *swr1*Δ *htz1*Δ strain (Figs 3A). Furthermore, Rad53 activation was also reduced in *swr1*Δ *htz1*Δ cells (S3A Fig). The reduced DSB induction could be explained by reduced AsiSI expression from the *pGAL1-10* promoter. Therefore, we evaluated AsiSI expression by anti-HA Western Blot (S3B Fig), including dilution series (S3C Fig). Semiquantitative analysis of Western Blots showed reduced AsiSI protein levels in *swr1*Δ *htz1*Δ cells (50% ± 30% S3D Fig), suggesting that changes in AsiSI expression may influence DSB induction. Moreover, H2A.Z and SWR1 could be involved in facilitating DSB induction by promoting DNA accessibility for the AsiSI restriction enzyme by changing local chromatin composition or architecture [65].

Taken together, the experiments shown in Fig 2 and 3 do not find supportive *in vivo* evidence for a role of H2A.Z as regulator of resection. Moreover, our data cautions that functions of H2A.Z in DNA damage may be difficult to assess genetically and may be occluded by general roles of H2A.Z in transcription and genome organisation [55–57,64].

### RSC and SWI/SNF complexes promote resection of deprotected telomeres

**Fig 4:**
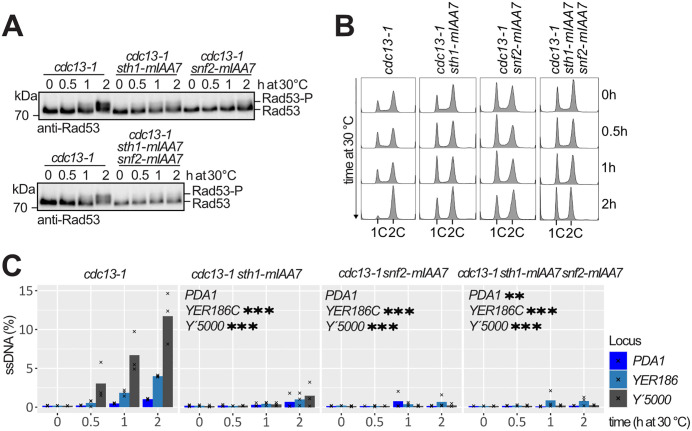
Resection of deprotected telomeres requires nucleosome eviction. **(A)** Dysfunctional nucleosome eviction leads to a reduced Rad53 activation after telomere deprotection in *cdc13-1* mutants: Western Blots detecting phosphorylated forms of Rad53 visible by gel shift show minor increases in levels of phosphorylated Rad53 after temperature shift to 30 °C of *cdc13-1 sth1-mIAA7, cdc13-1 snf2-mIAA7, cdc13-1 sth1-mIAA7 snf2-mIAA7* compared to *cdc13-1*. Data is representative of n = 2–3 biological replicates. **(B)** No cell cycle arrest of *cdc13-1* mutants deficient in nucleosome eviction after telomere deprotection: Mutant strains were grown at 23 °C, temperature shifted to 30 °C and samples taken at indicated timepoints. Total DNA content was measured by flow cytometry after SYTOX green staining. Data is representative of n = 2 biological replicates. **(C)** No extensive resection of deprotected telomeres of Chr V was measured for nucleosome eviction deficient mutants by quantitative amplification of ssDNA (QAOS): Yeast cultures of c*dc13-1, cdc13-1 sth1-mIAA7, cdc13-1 snf2-mIAA7* and *cdc13-1 sth1-mIAA7 snf2-mIAA7* were shifted to 30 °C and harvested at indicated timepoints. Accumulation of ssDNA was measured at *Y´5000*, *YER186C* and *PDA1* loci. Data is representative of n = 3 biological replicates. The significance of resection defects in the mutant strains compared to the *cdc13-1* control was calculated using a linear mixed-effects model. A single biological replicate of the *cdc13-1* control strain is the same as in Fig 1D.

Nucleosome eviction is a straight-forward mechanism by which nucleosome remodellers could promote resection through chromatin. Consistently, we previously observed that resection and nucleosome eviction were blocked at AsiSI-induced DSBs when we depleted the catalytic subunits of both RSC (Sth1) and SWI/SNF (Snf2) using an auxin-induced degron approach [21]. In contrast, short-range resection by MRX was not affected by RSC or SWI/SNF [66], suggesting that nucleosome eviction may be particularly critical for long-range resection. To ascertain whether RSC and/or SWI/SNF also act during resection of deprotected telomeres, we combined auxin-induced degradation of Sth1 and Snf2, with the *cdc13-1* mutation to generate *cdc13-1 sth1-mIAA7*, *cdc13-1 snf2-mIAA7* and *cdc13-1 sth1-mIAA7 snf2-mIAA7* strains. We verified by Western Blot that IAA addition to the yeast cultures led to efficient degradation of Sth1-mIAA7 or Snf2-mIAA7, respectively. Consistently, *sth1-mIAA7* and *snf2-mIAA7* mutant strains showed growth deficiencies in the presence of IAA (S4A-B Figs). In further experiments we induced Sth1 or Snf2 degradation 1h prior to temperature-shift to 30°C. Upon temperature induced telomere deprotection we observed G2/M cell cycle arrest and Rad53 activation in *cdc13-1* control strains, but all strains that lacked Sth1 or Snf2, such as *cdc13-1 sth1-mIAA7*, *cdc13-1 snf2-mIAA7* and *cdc13-1 sth1-mIAA7 snf2-mIAA7,* showed a reduced induction of Rad53 phosphorylation and no or delayed G2/M cell cycle arrest (Figs 4A, 4B). Therefore, we checked whether resection of deprotected telomeres was affected in strains that lacked RSC or SWI/SNF complexes. In the QAOS assay, we observed strong resection defects after telomere deprotection, whenever we depleted Sth1 or Snf2 (Fig 4C and S4C Fig). These data suggest that RSC and SWI/SNF complexes may play non-redundant roles in regulating long-range resection. Alternatively, we cannot exclude that RSC or SWI/SNF or both might act during the deprotection process itself or during resection of telomere ends bound by telomere binding proteins. Overall, these data suggest that the function of nucleosome evictors is not restricted to DSBs that occur within the genome, but that nucleosome evictors may act during resection after telomere deprotection, as well.

## Discussion

In this study, we revisited resection after *cdc13-1* mediatedtelomere deprotection in different budding yeast mutant strains. Using a degron approach, we found that Dna2 is involved in long-range resection after telomere deprotection (Fig 1), similar to what is known at DSBs at other genomic locations [7,8]. Our results thereby support a model in which Exo1 and Dna2 mediate long-range resection after telomere deprotection, similar as at other genomic locations. We therefore used the *cdc13-1* system to investigate the influence of chromatin factors on long-range resection. We did not find resection to be influenced by the absence of H2A.Z (*htz1*Δ *swr1*Δ double mutant) or by a dysfunctional INO80 complex (*arp8Δ,* Fig 2). In contrast, we found that inactivation of the main nucleosome evictors RSC or SWI/SNF had a strong effect on resection of deprotected telomeres, suggesting that nucleosome eviction is needed to allow for long-range resection or for telomere deprotection (Fig 4).

It has been unclear, whether the *cdc13-1* system is a useful model system not only for resection of deprotected telomeres, but for chromatin resection in general. To be a good model system for chromatin resection at least two criteria should be met. First, the resection machinery acting after telomere deprotection and at DSBs should be the same. Second, chromatin factors influencing the resection substrate also need to act similar at the different locations. Regarding the resection machinery, our data suggests that at least long-range resection nucleases Exo1 and Dna2 are both involved during resection of deprotected telomeres and show functional overlap, similar to what has been shown for general DSB resection [7,8]. Our parsimonious hypothesis is that long-range resection is sufficiently similar at deprotected telomeres and during genome-wide DSB resection.

With regard to chromatin, we reason that resection is likely influenced by the local chromatin structure, which will differ at different genomic locations. Telomeres have highly specialised chromatin: They contain a ssDNA overhang, which in budding yeast is bound by the CST complex. Furthermore, the adjacent telomeric DNA is bound by Rap1 and Sir proteins, which lead to transcriptional silencing of this locus [36–38,40–44]. In our model, telomeric deprotection is induced by the CST complex, but other telomeric binding proteins might still affect resection. It is therefore conceivable that initial resection upon telomere deprotection will differ from short-range resection occurring at genomic DSBs. In particular, the MRX complex promotes DNA end resection at double-strand breaks, whereas telomeric binding proteins like Rap1 together with Rif2 inhibit its activity at telomeres resulting in a protective rather than resection-promoting function [37,39,67–74]. However, long-range resection occurs over several kilobases of DNA and in our experiments, we measure resection over a window of 29 Kb (Fig 1D). Within this window, the DNA substrate is mostly genomic euchromatin. So, we hypothesise, that at deprotected telomeres the same chromatin remodellers are required for long-range resection like at other genomic locations. Consistently, we observed a similar involvement of nucleosome remodellers in the resection of deprotected telomeres and at genomic DSBs induced by restriction enzymes.

Most notably, we detected a strong defect in resection accompanied by reduced Rad53 phosphorylation and cell cycle arrest, whenever we degraded Snf2 (catalytic subunits of SWI/SNF) or Sth1 (catalytic subunit of RSC, Fig 4). This suggests that nucleosome eviction is required for long-range resection. Presumably the remodellers remove nucleosomes ahead of the resecting nuclease(s) and thereby break down the barrier to resection. Mechanistically, both complexes act as nucleosome evictors and one could therefore expect functional redundancy [75–77]. In contrast, we observed strong resection defects already in single *snf2-mIAA7* and *sth1-mIAA7* mutants, suggesting that RSC and SWI/SNF play non-redundant roles in promoting resection after telomere deprotection. Currently, it is unclear whether these non-redundant functions originate from differential recruitment/localisation of the two remodellers to distinct locations or whether they show specificity towards the nucleosomal substrate, for example euchromatin versus sub-telomeric (silenced) chromatin. We also cannot discriminate between functions in short- and long-range resection. Notably, an early role prior to resection of HO-induced DSBs has previously been suggested for the RSC complex [78]. In contrast, short-range resection by the MRX-Sae2 complex was found not to be affected upon depletion of RSC, suggesting it may have a late role during long-range resection [66]. Consistently, we showed that RSC and SWI/SNF complexes collectively promoted resection of restriction enzyme-induced DSB, but did only test the corresponding double mutant [21]. Furthermore, another study showed long-range resection defects in *snf5*Δ mutants [79], consolidating a function of SWI/SNF in nucleosome eviction. Future work will be required to disentangle the mechanistic roles of RSC and SWI/SNF during resection of genomic DSBs.

When interpreting these results, it is important to note that the QAOS assay measures single-stranded DNA formation at loci located several kilobases from the chromosome end. It therefore primarily reports on resection after telomere deprotection within adjacent chromatin rather than within the telomeric repeat tract itself. Consequently, our experiments do not directly assess whether nucleosome remodellers influence nucleolytic processing within telomeric DNA. It will therefore be interesting to determine in future work whether the RSC and SWI/SNF complexes also regulate processing of the telomeric repeat tract itself. Given that budding yeast telomeres are largely devoid of canonical nucleosomes, a potential role of these chromatin remodellers in promoting resection of such substrates would point to a function beyond canonical nucleosome eviction.

In addition to RSC and SWI/SNF, the nucleosome remodeller Fun30 has also been implicated in regulating long-range resection at enzyme-induced DSBs [15,16,24–28]. The molecular mechanism, by which Fun30 promotes resection is still under investigation. Analysis of its role in the *cdc13-1* telomere deprotection system will be reported elsewhere.

We also tested another possible mechanism for promoting chromatin resection and for overcoming the nucleosome barrier: nucleosome editing. Specifically, *in vitro* data suggested that H2A.Z incorporation into the break-surrounding chromatin could weaken the nucleosome-DNA interaction and allow long-range resection nucleases to proceed through these nucleosomes [22]. However, we failed to find support for a major contribution of nucleosome editing and SWR1 and INO80 complexes in chromatin resection at deprotected telomeres and at AsiSI-induced DSBs. At deprotected telomeres, we observed similar resection in *cdc13-1* and *cdc13-1 swr1*Δ *htz1*Δ cells. Using the AsiSI system, we were unable to conclude about the role of H2A.Z or the SWR1-complex as AsiSI-dependent DSB induction was hampered in this genetic background. Our experiments highlight that the *cdc13-1* system is of particular use in genetic backgrounds for which protein-expression-dependent DSB-induction is not possible.

## Methods

### Budding yeast strains and culture

All budding yeast strains used in this study are derived from W303 background and are listed in S1Table. Gene deletions and tags were introduced using a PCR-based protocol [80]. Integrative plasmids were linearized before transformation. Genotyping was verified via colony PCR. The temperature sensitive *cdc13-1* mutant in W303 background was obtained from Brian Luke and corresponds to the allele *cdc13-P371S* [81]. All *cdc13-1* yeast strains were grown at 23 °C unless indicated otherwise, whilst all other strains were grown at 30 °C. In general, YP-medium supplemented with 2% glucose (YPD) was used for cultivation. For experiments using *pGAL1-10*-*AsiSI*, YP-medium supplemented with 2% raffinose (YPR) was used for cultivation and conditional expression was achieved by addition of galactose (2% final concentration). For M-phase arrest, cultures were treated with 5 µg/mL nocodazole (Sigma, M1404) for 3 h before the start of the experiment. Degradation of mIAA7-tagged proteins (Dna2, Sth1, Snf2) was achieved by treatment with 1 mM IAA (Sigma, I3750) for 1 h before starting the experiment.

### Growth assays

Saturated yeast cultures, grown at 23 °C due to the *cdc13-1* mutation, were diluted to 1 OD600 and six five-fold serial dilutions were spotted onto YPD plates (2% glucose). Photographs were taken after 48 h incubation at the indicated temperatures. To test the influence of degradation of mIAA7-tagged proteins, YPD plates containing 1 mM IAA (Sigma, I3750) were used in addition to YPD control plates. For each experiment, biological replicates were obtained each including two technical replicates for each condition.

### Flow cytometry

Yeast cells were pelleted and resuspended in fixation buffer (70% ethanol 50 mM Tris-HCl, pH 8.0) to be stored at 4 °C for at least overnight. Fixed cells were washed with 50 mM Tris-HCl, pH 8.0 and then treated with 520 µL RNase A solution consisting of 500 µL 50 mM Tris-HCl, pH 8.0 and 20 µL RNase A (Sigma, R4875; 10 mg/mL in 10 mM Tris-HCl, pH 8.0, 10 mM MgCl_2_) for at least 4 h at 37 °C. Cells were pelleted and treated with 220 µL Proteinase K solution consisting of 200 µL 50 mM Tris-HCl, pH 8.0 and 20 µL Proteinase K (Sigma, P2308; 20 mg/mL in 10 mM Tris-HCl, pH8.0) for 30 min at 50 °C. Finally, cells were resuspended in 500 µL 50 mM Tris-HCl, pH 8.0 and sonicated. The final cell suspensions were diluted 1:20 with SYTOX Green solution (Invitrogen, S7020, 1 µM in 50 mM Tris-HCl, pH 8.0) and measured using a MACSquant Analyzer 10 Flow Cytometer (Milteny Biotec). Data containing 30000 events per sample were analysed and plotted using FlowJo as previously described [82] (version 10.10.0) (FlowJo LLC).

### SDS-PAGE and Western Blot

2 × 10^7^ yeast cells (corresponding to 1 mL of cell suspension at OD600 = 1) were harvested at the indicated timepoints and snap-frozen in liquid nitrogen. For alkaline lysis, yeast pellets were resuspended in 1 mL of ice-cold water, 150 µL 1.85 M NaOH and 7.5% β-mercaptoethanol and incubated on ice for 15 min. 150 µL of 55% trichloroacetic acid (TCA, Roth, 8789.2) were added and samples were incubated for 10 min on ice before centrifugation. Pellets were resuspended in 50 µL HU buffer (8 M urea, 5% SDS, 200 mM Tris-HCl pH 6.8, 1.5% DTT, traces of bromophenol blue) and heated for 10 min at 65 °C.

Samples were run on NuPAGE 3–8% Tris-Acetate acrylamide gels (Invitrogen, 30131968) with MOPS buffer (50 mM MOPS, 50 mM Tris base, 0.1% SDS, 1 mM EDTA) for 1.5 h at 180 V. To resolve Rad53 phospho-shifts, samples were run on 10% acrylamide gels in SDS buffer (25 mM Tris base, 192 mM glycine, 0.1% SDS) for 2.5 h at 150 V at 4 °C.

For Western Blotting, proteins were transferred to a nitrocellulose membrane using transfer buffer (48 mM Tris base, 39 mM glycine, 0.0375% SDS, 20% methanol) at 4 °C for 90 min at 90 V. Incubation with primary antibody was done overnight at 4 °C while shaking. Membranes were washed three times for 5 min with western wash buffer (0.2% NP-40 in TBS). Secondary HRP-coupled antibodies were incubated for 2 h at room temperature. Membranes were washed 3 times for 10 min and chemiluminescence was detected using Pierce ECL reagents (Thermo Fisher Scientific, 32106) and ChemiDoc MP imaging system (Bio-Rad). The antibodies used in this study are listed in S3 Table. All dilutions were performed in Superblotto solution (2.5% skim milk powder, 0.5% BSA, 0.5% NP-40, 0.1% Tween in TBS). Images of the uncropped Western Blot images are provided in S1 Raw Images.

For semi-quantitative Western Blot analysis three 2-fold serial dilutions were blotted. Background subtracted signal intensities of the protein of interest (AsiSI-3HA) and the loading control (Cdc48) were quantified using Fiji (ImageJ 1.54p) and their ratio plotted.

### Quantitative amplification of ssDNA (QAOS)

Telomere deprotection of *cdc13-1* strains was induced by temperature-shift to 30°C. With the exception of the long time-course experiment of Fig 2E, samples were taken 30, 60 and 120 min after temperature shift. For Fig 2E, samples were taken 2, 4 and 6 h after temperature shift. Approximately 1.6 × 10^9^ yeast cells (corresponding to 80 mL of cell suspension at OD600 = 1) were harvested, washed in TE20 buffer (10 mM Tris pH 8.0, 20 mM EDTA) and frozen in liquid nitrogen to be stored at −80 °C until further processing. The pellets were resuspended in 1.8 mL freshly prepared Buffer Y1 (1 M Sorbitol, 0.1 M EDTA, 14 mM β-mercaptoethanol) supplemented with 40 µL Zymolyase 20T (100 mg/mL in n2 buffer (1.2 M Sorbitol, 50 mM EDTA, 50 mM Sodium citrate phosphate buffer pH = 5,6 (2.68% (w/v) Na_2_HPO_4_, 0.92% (w/v) citric acid))). After incubation for 40 min at 37 °C with regular inversion, effective spheroplasting (>90%) was monitored by microscopic examination and samples were centrifuged at 4 °C. Spheroplasts were resuspended in 200 µL breaking buffer (2% Triton X 100, 1% SDS, 100 mM NaCl, 10 mM Tris Cl pH 8.0, 1 mM EDTA) and transferred to a phase-lock-tube. 200 µL phenol/chloroform/isoamyl alcohol was added and samples were incubated for 15 min with regular inversion. 200 µL TE buffer (10 mM Tris pH 8.0, 1 mM EDTA) was added, samples mixed by inversion and centrifuged (5 min, 1400 rpm). The aqueous layer was transferred to a new tube and 1 mL of 100% ethanol added. After inversion, samples were centrifuged (3 min, 1400 rpm) and pellets were resuspended in 400 µL TE buffer. 30 µl DNase-free RNase A (1 mg/mL) was added and samples were incubated for 5 min at 37 °C. 5 µL Proteinase K (1 mg/mL) was added and samples incubated for 1 h at 37 °C. gDNA was precipitated by adding 10 µL ammonium acetate 4M and 1 mL ethanol 100%. The DNA pellet was dried and resuspended in 40 µl TE20 buffer for 1 h at RT.

For QAOS ssDNA tagging reaction, 25 ng of gDNA (0.5 ng/µL final concentration) were treated with 25 µL Q5 High-Fidelity 2X Master Mix (NEB, M0942), 2.5 µL of the corresponding tagging oligo (final concentration 500 nM) and 17.5 µL sterile water. *PAC2* locus is used as a control and does not require tagging, therefore a mock tagging reaction was performed with sterile water instead of tagging oligos. Tagging reactions were performed at 40 °C 5 min and 72 °C 5 min. After tagging, qPCR was performed to determine ssDNA signal at telomeres by adding 2.5 µL forward oligo (10 µM), 2.5 µL reverse oligo (10 µM), 0.2 µL Blue Dye (100x, 172555 BioRad) and 0.5 µL dsGreen (100 x, 11010 Lumiprobe). Each sample was divided in 3 technical replicates (10 µL) in a 384 multiwell plate and run in a LightCycler480 (Roche) with the following settings: activation at 98 °C for 3 min and 40 amplification cycles of 98°C for 10 s, 64 °C for 5 s and 72 °C for 10 s. To ensure specific amplification, melting curve analysis was performed (65 °C – 97 °C ramp rate 0.11 °C/s). Tagging and amplification oligos used in this study are listed in S2 Table.

Ct values were calculated via 2^nd^ derivative analysis and are listed in S5 Table. The ssDNA/dsDNA standard curves were used to translate the Ct values into percentage of ssDNA. ssDNA was generated by boiling (4 cycles: 98 °C for 7 min and 25 °C for 30 s) and mixed with different amounts of dsDNA. For *PAC2* locus, the actual DNA amount of the sample was calculated from the measured Ct value and the dilution series to determine the correction factor for normalisation of the ssDNA percentage. The determined ssDNA amount was multiplied by the correction factor and plotted. Using the same strains, a shift in experimental values of the 2h samples was observed when comparing short and long time course experiments of Fig 2D and Fig 2E, which were conducted with a temporal gap of 4 months and by two different researchers.

Significant differences of tested mutants compared to the *cdc13-1* control strain were determined using a linear mixed-effects model implemented in RStudio using the lme4 package. Specifically, we assumed linear growth of ssDNA over the time course of the experiment and conducted linear regression, as this fitted best to the observed increase in ssDNA. The linear mixed-effects model considers the different timepoints and yeast strains as fixed effects, while variations between biological replicates are considered random effects. The formula: lmer(percentage ~ time0 + time0:yeast_strain + (0 + time0 | replicate) was used, assuming all strains have the same amount ssDNA at the 0 h time point, but then accumulate ssDNA with different speeds. The calculated mean slope values for each strain are listed in S4 Table. The significances between the *cdc13-1* control strain and the mutants are indicated with * in the figures: p-value < 0.05 corresponds to *, p-value < 0.01 corresponds to ** and p-value < 0.001 corresponds to ***. qPCR source data are provided in S5 Table.

### Analysis of DSB resection by Strand-specific Sequencing

Exponentially growing cells containing *pGAL1-10 AsiSI* grown in YP-Raffinose were treated with nocodazole (5 µg/mL) for 3 h to induce mitotic cell cycle arrest. After taking samples for the 0 h timepoint, galactose was added to 2% final concentration to induce AsiSI expression and DSB induction. Samples for flow cytometry, Western Blotting and Nanopore Sequencing were collected for each timepoint in addition to the samples for strand-specific sequencing. Cells were processed as previously described [21]. Briefly, 100 mL samples were crosslinked with para-formaldehyde (1% final concentration) for 16 min before quenching with glycine. For each timepoint, 1.6 × 10^9^ yeast cells (corresponding to 80 mL of cell suspension at OD600 = 1) were harvested, washed twice in cold PBS and snap-frozen. Cell lysis was performed by resuspension in 800 μl lysis buffer (50 mM HEPES KOH pH 7.5, 150 mM NaCl, 1 mM EDTA, 1% Triton X-100, 0.1% Na-deoxycolate, 0.1% SDS) supplemented with protease inhibitors (10 mM Pefabloc SC (Sigma-Aldrich) and 1 cOmplete protease inhibitor tablet (Roche) per 50 mL lysis buffer). Silica beads were added and lysis was performed using a beat beater (MM301, Retsch GmbH) for 6 cycles of 3 min ON/ 3 minutes OFF. After centrifugation, the insoluble chromatin fraction was resuspended in 1 mL cold lysis buffer containing protease inhibitors and sonified using Diagenode Bioruptor UCD-200 to shear the DNA in 200–500 bp fragments. After centrifugation, the cleared lysate was diluted with 1 mL (1:1) lysis buffer containing protease inhibitors. 20 µL (1%) was taken as input sample to analyse the loss of DNA around the AsiSI induced DSB sites. 100 µL ChIP elution buffer (50 mM Tris-HCl pH 7.5, 10 mM EDTA pH 8.0, 0.1% SDS) was added together with 5 µL RNase A (10 mg/mL) and incubated for 45 min at 37 °C. To remove proteins, 20 µL Proteinase K (20 mg/mL) was added and samples were incubated for 1 h at 42 °C. Protein crosslinks were reversed incubating at 65 °C for an overnight. DNA fragments were purified using the QIAquick PCR purification kit 250 (QIAGEN 28106) and eluted in 25 µL water.

For strand-specific library preparation, the Accel-NGS 1S Plus Library Kit was used accordingly to the manufacturer’s instruction, treating. 2 ng of DNA and 14 cycles of library amplification. For clean-up, SPRIselect beads were used. Paired-end Illumina sequencing with 75 cycles per read was performed at the sequencing facility of the MPIB NGS facility (Martinsried, Germany). The experiment was performed twice showing similar results.

For data analysis, bowtie2 was used to map reads to the *S.cerevisiae* R64 (*sacCer3)* genome and multiple mapping reads were excluded using SAMtools. Using RStudio (version 4.2.3) coverage was adjusted to the same amount for all samples by randomly excluding excessive reads. Forward and reverse coverage was calculated separately, samples were normalised to the 0 h timepoint and the mean coverage around 13 AsiSI sites [21] was plotted in a window over 20 kb. The NGS data generated is deposited in the NCBI GEO database under accession code GSE325557.

### Analysis of DSB induction by Nanopore Sequencing

1.6 × 10^9^ yeast cells (corresponding to 80 mL of cell suspension at OD600 = 1) were harvested, washed in TE20 (10 mM Tris pH 8.0, 20 mM EDTA) and snap frozen in liquid nitrogen to be stored at −80 °C until further processing. gDNA was extracted analogously to QAOS. Before library preparation, an additional purification step using AMPure XP beads (A63881 Beckman Coulter) was performed by adding 1.8 times beads volume, incubation for 5 min, precipitation with a magnetic rack and 2 washes with 80% ethanol before eluting with water for 10 min.

Libraries were prepared accordingly to the manufacturer’s instructions for the Native Barcoding Kit 24 V14 (SQK-NBD114.24). Briefly, DNA repair and end-prep was performed by treating between 800–1000 ng of gDNA with FFPE DNA Repair Mix (NEB: M6630S and E6622A) and Ultra II End-Prep Enzyme Mix (NEB: E7646A and E7647A), 5 min at 20 °C and 5 min at 65 °C. Then, native barcode ligation was performed to allow for multiplexing by ligating End-prepped DNA with Native Barcodes (NB01-24) using Blunt/TA Ligase Master Mix (NEB: M0367S) for 20 min at RT. Finally, barcoded DNA was ligated to Native Adapter using Quick T4 DNA Ligase (NEB: M2200S and B2200S) for 20 min at RT. Nanopore Sequencing was performed using R10 Flowcells in a MinION Mk1B sequencer with standard settings. Basecalling was performed using Dorado basecaller (Dorado 7.9.8) integrated in MinKNOW using high-accuracy basecalling model (dna_r10.4.1_e8.2_400bps_5khz). Unaligned bam files were mapped to the *S.cerevisiae* R64 (*sacCer3)* reference genome using Epi2me workflow “*wf alignment*” using default settings. Mean coverage of the 2 bp DNA sequence (AT of the AsiSI cutsite) was determined, as this sequence is lost from Nanopore reads upon cutting. For total coverage, the mean value of two genomic windows of 5 kb to the left and right the DSB were determined. To avoid artefacts related to DNA end resection, the windows were displaced 5 kb from the centre of the AsiSI cutsite, thus covering the area between 5 and 10 kb from the DSB. The percentage of uncut DNA is given by dividing the mean coverage of the 2 bp AsiSI sequence by the mean coverage of the windows around the DSB. The cutting efficiency is 1 minus the uncut DNA proportion. Both biological repetitions of the strand-specific sequencing experiments were analysed via Nanopore Sequencing to measure the corresponding cutting efficiencies.

### Statistical and computational analysis

All biological replicates were generated by either using a different clone with the same genotype or independent cultivation of the same yeast strain of clonal origin. For QAOS experiments the mean of three technical replicates was calculated for each biological replicate. Means across biological replicates were obtained and a linear mixed-effects model was used to test for significance. Significance is indicated in the respective figures: p-value < 0.05 corresponds to *, p-value < 0.01 corresponds to ** and p-value < 0.001 corresponds to ***. All code used in this study was done in RStudio R4.2.3 using the packages BSgenome.Scerevisiae.NIH.sacCerW303, readxl, writexl, lme4, pbkrtest, lmerTest, emmeans, ggsignif, lmerTest, GenomicRanges, GenomicAlignments, rtracklayer, Rsamtools, dplyr, tidyr, tidyverse, reshape2, parallel, grid, gridExtra, cowplot, ggplot2 and Cairo (S1 File). S6 Table serves as template for structuring the raw data.

## Supporting information

S1 Fig Dna2 is involved in resection of deprotected telomeres. **(A)** Rad53 activation after telomere deprotection in *cdc13-1* mutants: Western Blots detecting phosphorylated forms of Rad53 visible by gel shift show increasing levels of phosphorylated Rad53 after temperature shift to 30 °C of the *cdc13-1* mutant, but not of WT cells. Data is representative of n = 1 biological replicate. **(B)** *cdc13-1* cells do not activate Rad53 at permissive temperature: Western Blots detecting phosphorylated forms of Rad53 visible by gel shift show no phosphorylated Rad53 for *cdc13-1* and WT incubated at 23 °C. Data is representative of n = 1 biological replicate. **(C)** Standard curves of the qPCRs used for QAOS analysis: For *PAC2* control locus a 10-fold dilution series was measured and used to normalise for differences in DNA loading. For *Y´5000, YER186C* and *PDA1* loci, qPCR efficiency was measured by a ssDNA/dsDNA dilution series. The standard curves were used for translation of the measured Ct values into the corresponding ssDNA percentages. **(D)** Resection of deprotected telomeres of Chr V was measured by quantitative amplification of ssDNA (QAOS): One *cdc13-1* culture was shifted to 30 °C, whilst the other was kept at 23 °C and harvested at indicated timepoints. Accumulation of ssDNA was measured at *Y´5000, YER186C* and *PDA1* loci. Data is representative of n = 2 biological replicates. **(E)** Linear mixed-effects model of QAOS experiment in Fig 1D: The significance of the difference of increasing ssDNA levels with time after temperature shift to 30 °C was calculated for *cdc13-1 exo1∆, cdc13-1 sgs1∆ and cdc13-1 exo1∆ sgs1∆* compared to the *cdc13-1* control strain. Slope values are provided in S4 Table. **(F)** Dna2-mIAA7 is degraded upon addition of auxin: Anti-Flag Western Blot detecting levels of Dna2-mIAA7-3Flag in *cdc13-1 dna2-mIAA7, cdc13-1 dna2-mIAA7 exo1∆* and *cdc13-1 dna2-mIAA7 sgs1∆* strains before and after 1 h of IAA treatment (final concentration 1mM). Asterisk denotes a cross-reactive band. Data is representative of n = 1 biological replicate. **(G)** Growth defects of *dna2-mIAA7* strains on IAA plates indicate successful Dna2 degradation: Five-fold serial dilutions of WT, *cdc13-1, cdc13-1 dna2-mIAA7, cdc13-1 dna2-mIAA7 exo1∆* and *cdc13-1 dna2-mIAA7 sgs1∆* were spotted on YPD and YPD + IAA (1mM) plates and incubated for 48 h at indicated temperatures. Data is representative of n = 2−4 biological replicates. **(H)** Linear mixed-effects model of QAOS for *dna2-mIAA7* strains in Figure 1G: As in (E) but of *cdc13-1, cdc13-1 dna2-mIAA7, cdc13-1 dna2-mIAA7 sgs1∆* and *cdc13-1 exo1∆ dna2-mIAA7*. Slope values are provided in S4 Table. **(I)** Linear mixed-effects model of QAOS for the *cdc13-1 exo1∆ dna2-mIAA7* mutant in Fig 1D and G compared to *cdc13-1 exo1∆* and *cdc13-1 dna2-mIAA7.* Slope values are provided in S4 Table.
(TIFF)

S2 Fig Nucleosome editing does not affect resection of deprotected telomeres. **(A)** Linear mixed-effects model of QAOS experiment in Fig 2D for chromatin remodeller mutants: The significance of the difference of increasing ssDNA levels with time after temperature shift to 30°C was calculated for *cdc13-1 arp8∆, cdc13-1 swr1∆ htz1∆* and *cdc13-1 arp8∆ swr1∆ htz1∆* compared to the *cdc13-*1 control strain. Slope values are provided in S4 Table. **(B)** Linear mixed-effects model of QAOS experiment in Fig 2E for chromatin remodeller mutants: As in (A) but for 0, 2, 4 and 6 h. Slope values are provided in S4 Table.
(TIFF)

S3 Fig H2A.Z is required for AsiSI-dependent DSB induction. (A) Rad53 activation after DSB induction of *pGal-AsiSI* strains: Western Blots detecting phosphorylated forms of Rad53 visible by gel shift show increasing levels of phosphorylated Rad53 after DSB induction for *pGal-AsiSI, pGal-AsiSI arp8∆, pGal-AsiSI swr1∆ htz1∆* and *pGal-AsiSI arp8∆ swr1∆ htz1∆*. Data is representative of n = 2 biological replicates. **(B)** Reduced AsiSI-3HA expression in *swr1∆ htz1∆* mutants: Anti-HA Western Blot detecting levels of 3HA-tagged AsiSI in *pGal-AsiSI, pGal-AsiSI arp8∆, pGal-AsiSI swr1∆ htz1∆* and *pGal-AsiSI arp8∆ swr1∆ htz1∆* after the indicated time points. Asterisk denotes a cross-reactive band. Data is representative of n = 2 biological replicates. **(C)** AsiSI-3HA expression in *swr1∆ htz1∆* mutant samples compared to the *pGal-AsiSI* control strain with dilutions: Anti-HA Western Blot detecting levels of 3HA-tagged AsiSI after the indicated time points. The anti-Cdc48 Western Blot serves as loading control. Data is representative of n = 4 biological replicates. **(D)** Semiquantitative analysis of Western Blot dilution series of (C) show reduced AsiSI expression in the *swr1∆ htz1∆* mutant strain: Mean signal intensities of 3HA-tagged AsiSI expression normalised by the corresponding signal intensity of the Cdc48 loading control of 4 biological replicates is shown for a 2-fold dilution series for *pGal-AsiSI* and *pGal-AsiSI swr1∆ htz1∆* after the indicated timepoints.
(TIFF)

S4 Fig Resection of deprotected telomeres requires nucleosome eviction. **(A)** Sth1 and Snf2 are degraded upon addition of auxin: Anti-Flag Western Blot detecting levels of 3Flag-tagged Sth1 and Snf2 in *cdc13-1 sth1-mIAA7, cdc13-1 snf2-mIAA7* and *cdc13-1 sth1-mIAA7 snf2-mIAA7* before and after 1 h of IAA treatment (final concentration 1 mM). The anti-Pgk1 Western Blot serves as loading control. Data is representative of n = 2 biological replicates. **(B)** Growth defects of *sth1-mIAA7, snf2-mIAA7* and *sth1-mIAA7 snf2-mIAA7* strains on IAA plates indicate successful degradation: Five-fold serial dilutions of WT, *cdc13-1, cdc13-1 sth1-mIAA7, cdc13-1 snf2-mIAA7* and *cdc13-1 sth1-mIAA7 snf2-mIAA7* were spotted on YPD and YPD + IAA (1 mM) and incubated for 48 h at indicated temperatures. Data is representative of n = 2 biological replicates. **(C)** Linear mixed-effects model of QAOS experiment in Fig 4C for nucleosome evictor mutants: The significance of the difference of increasing ssDNA levels with time after temperature shift to 30 °C was calculated for *cdc13-1 sth1-mIAA7, cdc13-1 snf2-mIAA7* and *cdc13-1 sth1-mIAA7 snf2-mIAA7* compared to the *cdc13-*1 control strain. Slope values are provided in S4 Table.
(TIFF)

S1 Raw Images Uncropped western blots. **(A–K)** Uncropped Western Blot images. Rectangles represent the selected areas for the main figures. (A) Anti-Rad53 Western Blots corresponding to Fig 1B. (B) Anti-Rad53 Western Blots corresponding to Fig 1E. (C) Anti-Rad53 Western Blot corresponding to S1A Fig. (D) Anti-Rad53 Western Blot corresponding to S1B Fig. (E) Anti-Flag Western Blot corresponding to S1F Fig. (F) Anti-Rad53 Western Blots corresponding to Fig 2B. (G) Anti-Rad53 Western Blots corresponding to S3A Fig. (H) Anti-HA Western Blot corresponding to S3B Fig. (I) Anti-HA and anti-Cdc48 Western Blots corresponding to S3C Fig. (J) Anti-Rad53 Western Blots corresponding to Fig 4A. (K) Anti-Flag and Anti-Pgk1 Western Blot corresponding to S4A Fig.
(TIFF)

S1 Table Yeast strains. (XLSX)

S2 Table Oligonucleotides. (XLSX)

S3 Table Antibodies. (XLSX)

S4 Table Slope values of mixed-effects models. (XLSX)

S5 Table QAOS raw data. (XLSX)

S6 Table Excel template for QAOS analysis by R. (XLSX)

S1 File R-script for QAOS analysis. (R)
