# Supplementary figures and images for: A role for nucleosome remodellers during resection of deprotected telomeres in yeast
Source: PLoS One. 2026 Jul 10;21(7):e0352656. doi: 10.1371/journal.pone.0352656 (PMC13353985; doi:10.1371/journal.pone.0352656)

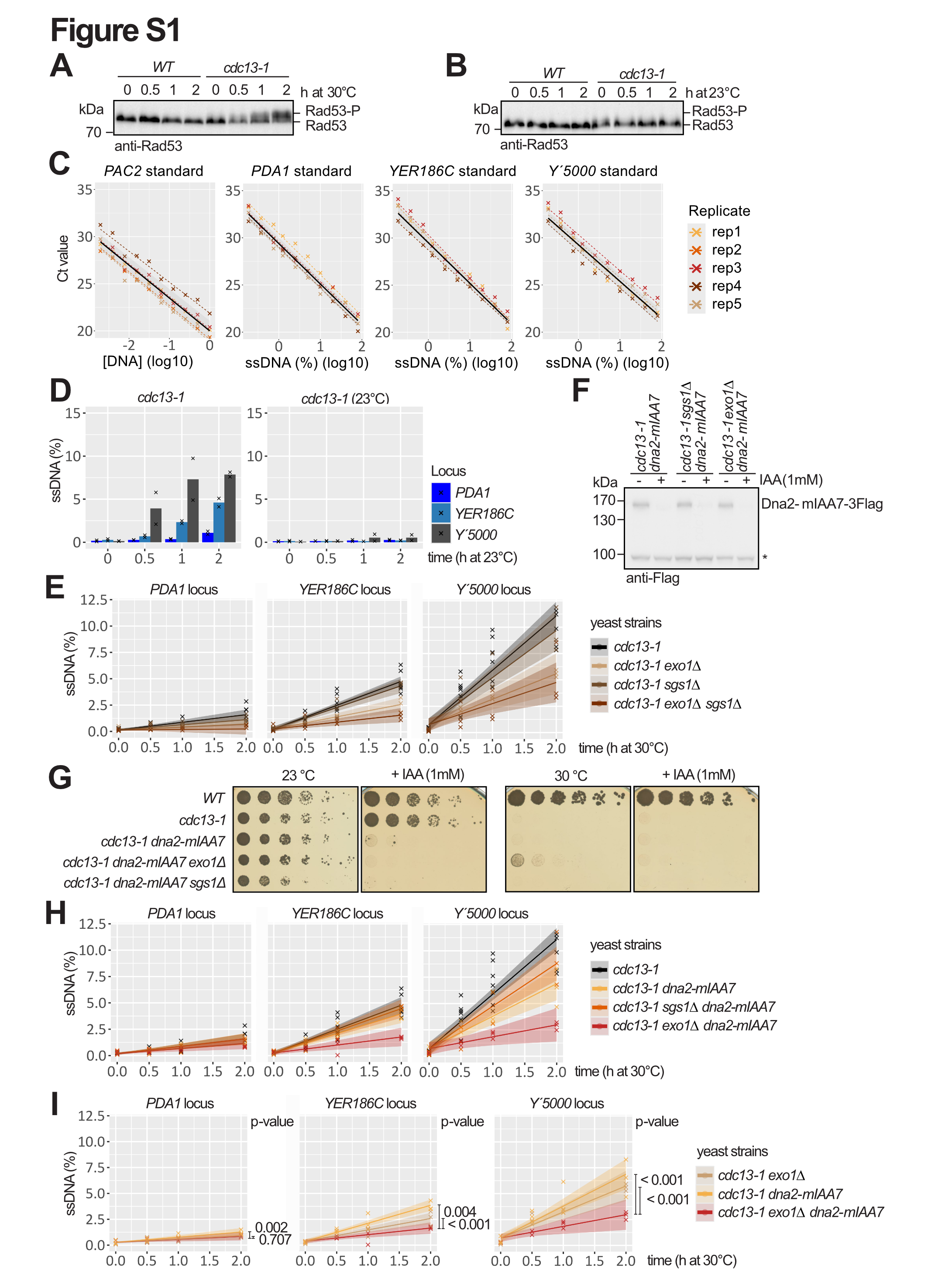

Supplement: S1 Fig — (A) Rad53 activation after telomere deprotection in cdc13-1 mutants: Western Blots detecting phosphorylated forms of Rad53 visible by gel shift show increasing levels of phosphorylated Rad53 after temperature shift to 30 °C of the cdc13-1 mutant, but not of WT cells. Data is representative of n = 1 biological replicate. (B) cdc13-1 cells do not activate Rad53 at permissive temperature: Western Blots detecting phosphorylated forms of Rad53 visible by gel shift show no phosphorylated Rad53 for cdc13-1 and WT incubated at 23 °C. Data is representative of n = 1 biological replicate. (C) Standard curves of the qPCRs used for QAOS analysis: For PAC2 control locus a 10-fold dilution series was measured and used to normalise for differences in DNA loading. For Y´5000, YER186C and PDA1 loci, qPCR efficiency was measured by a ssDNA/dsDNA dilution series. The standard curves were used for translation of the measured Ct values into the corresponding ssDNA percentages. (D) Resection of deprotected telomeres of Chr V was measured by quantitative amplification of ssDNA (QAOS): One cdc13-1 culture was shifted to 30 °C, whilst the other was kept at 23 °C and harvested at indicated timepoints. Accumulation of ssDNA was measured at Y´5000, YER186C and PDA1 loci. Data is representative of n = 2 biological replicates. (E) Linear mixed-effects model of QAOS experiment in Fig 1D: The significance of the difference of increasing ssDNA levels with time after temperature shift to 30 °C was calculated for cdc13-1 exo1∆, cdc13-1 sgs1∆ and cdc13-1 exo1∆ sgs1∆ compared to the cdc13-1 control strain. Slope values are provided in S4 Table. (F) Dna2-mIAA7 is degraded upon addition of auxin: Anti-Flag Western Blot detecting levels of Dna2-mIAA7-3Flag in cdc13-1 dna2-mIAA7, cdc13-1 dna2-mIAA7 exo1∆ and cdc13-1 dna2-mIAA7 sgs1∆ strains before and after 1 h of IAA treatment (final concentration 1mM). Asterisk denotes a cross-reactive band. Data is representative of n = 1 biological replicate. (G) Growt [file pone.0352656.s001.tiff]

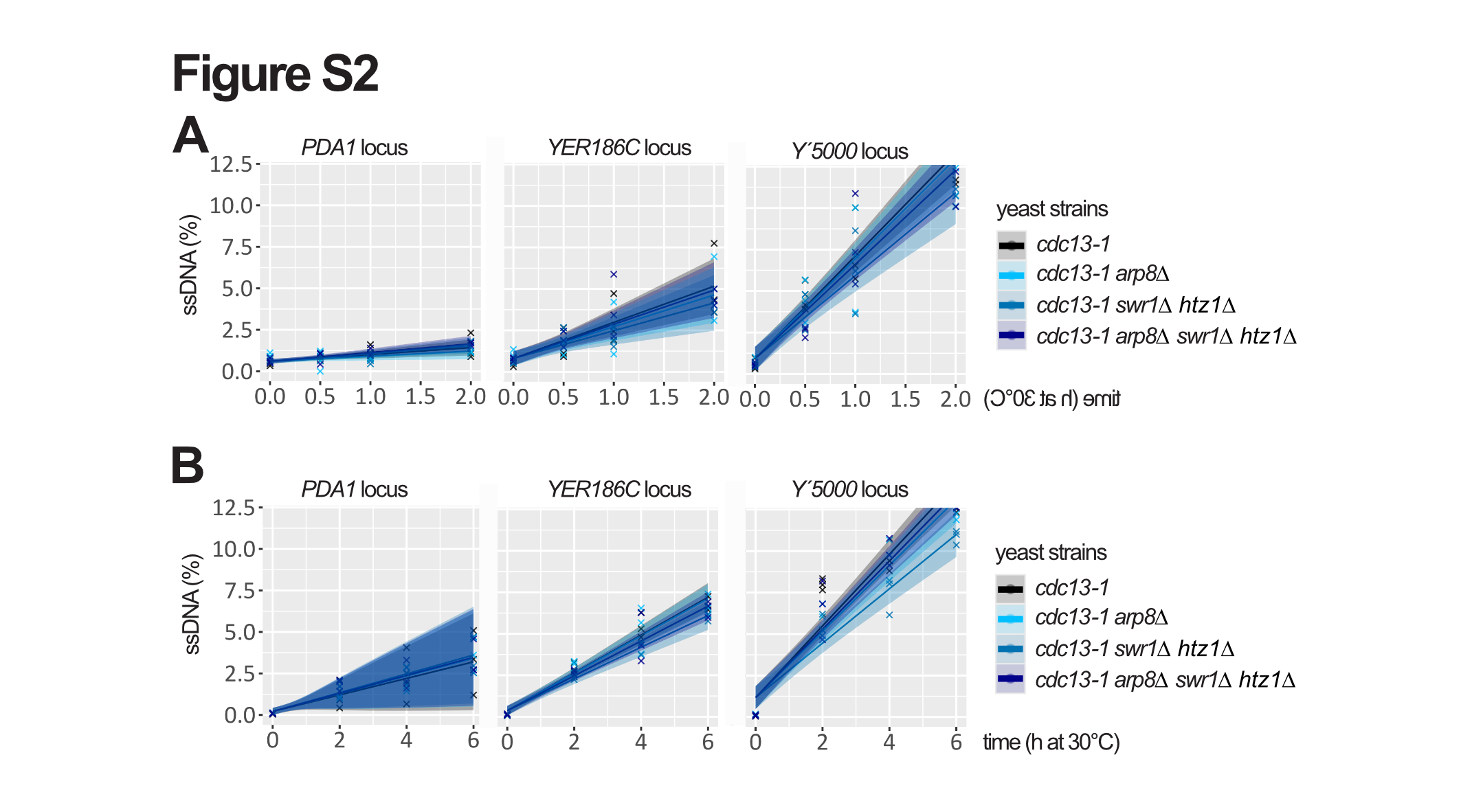

Supplement: S2 Fig — (A) Linear mixed-effects model of QAOS experiment in Fig 2D for chromatin remodeller mutants: The significance of the difference of increasing ssDNA levels with time after temperature shift to 30°C was calculated for cdc13-1 arp8∆, cdc13-1 swr1∆ htz1∆ and cdc13-1 arp8∆ swr1∆ htz1∆ compared to the cdc13-1 control strain. Slope values are provided in S4 Table. (B) Linear mixed-effects model of QAOS experiment in Fig 2E for chromatin remodeller mutants: As in (A) but for 0, 2, 4 and 6 h. Slope values are provided in S4 Table. (TIFF) [file pone.0352656.s002.tiff]

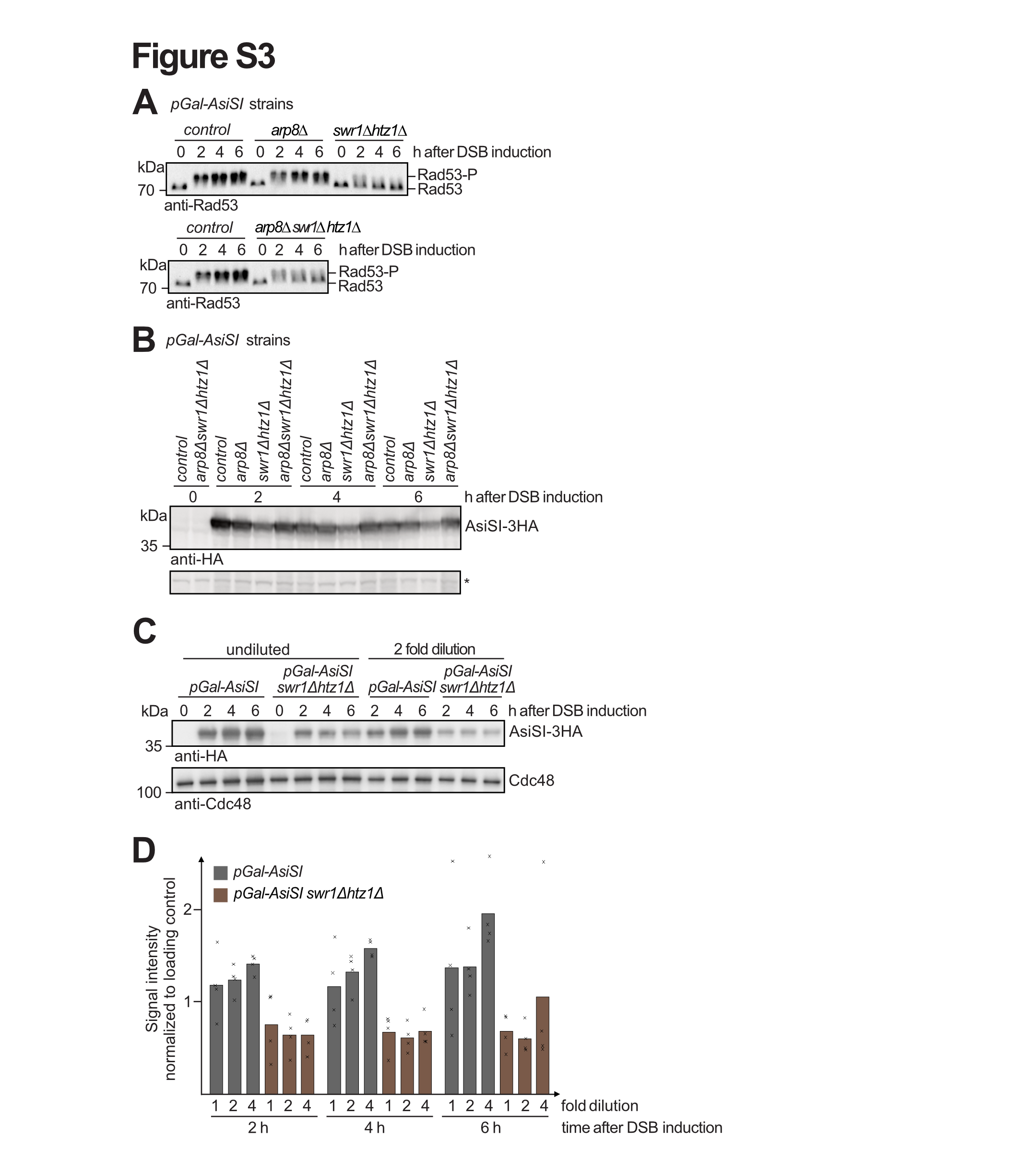

Supplement: S3 Fig — (A) Rad53 activation after DSB induction of pGal-AsiSI strains: Western Blots detecting phosphorylated forms of Rad53 visible by gel shift show increasing levels of phosphorylated Rad53 after DSB induction for pGal-AsiSI, pGal-AsiSI arp8∆, pGal-AsiSI swr1∆ htz1∆ and pGal-AsiSI arp8∆ swr1∆ htz1∆. Data is representative of n = 2 biological replicates. (B) Reduced AsiSI-3HA expression in swr1∆ htz1∆ mutants: Anti-HA Western Blot detecting levels of 3HA-tagged AsiSI in pGal-AsiSI, pGal-AsiSI arp8∆, pGal-AsiSI swr1∆ htz1∆ and pGal-AsiSI arp8∆ swr1∆ htz1∆ after the indicated time points. Asterisk denotes a cross-reactive band. Data is representative of n = 2 biological replicates. (C) AsiSI-3HA expression in swr1∆ htz1∆ mutant samples compared to the pGal-AsiSI control strain with dilutions: Anti-HA Western Blot detecting levels of 3HA-tagged AsiSI after the indicated time points. The anti-Cdc48 Western Blot serves as loading control. Data is representative of n = 4 biological replicates. (D) Semiquantitative analysis of Western Blot dilution series of (C) show reduced AsiSI expression in the swr1∆ htz1∆ mutant strain: Mean signal intensities of 3HA-tagged AsiSI expression normalised by the corresponding signal intensity of the Cdc48 loading control of 4 biological replicates is shown for a 2-fold dilution series for pGal-AsiSI and pGal-AsiSI swr1∆ htz1∆ after the indicated timepoints. (TIFF) [file pone.0352656.s003.tiff]

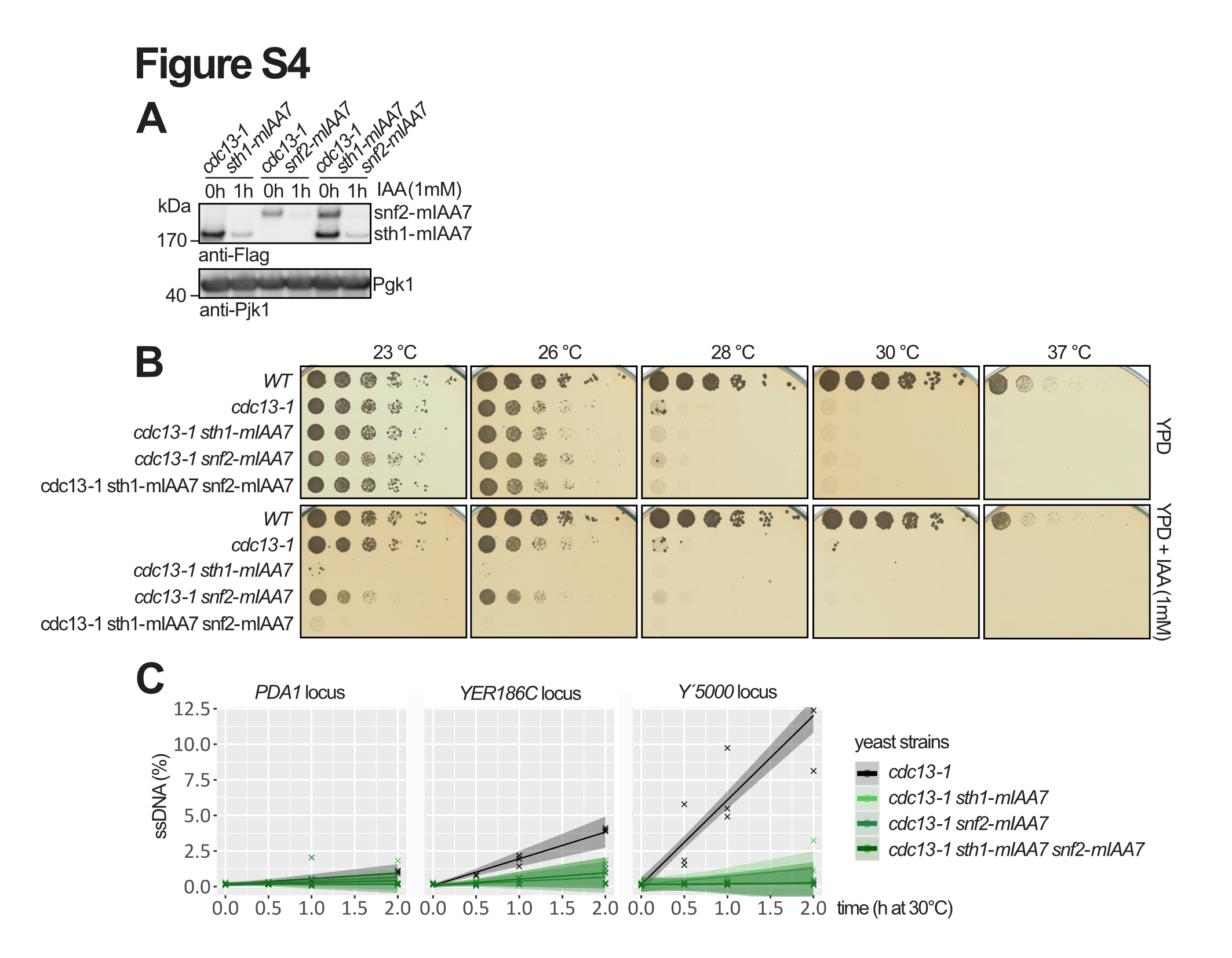

Supplement: S4 Fig — (A) Sth1 and Snf2 are degraded upon addition of auxin: Anti-Flag Western Blot detecting levels of 3Flag-tagged Sth1 and Snf2 in cdc13-1 sth1-mIAA7, cdc13-1 snf2-mIAA7 and cdc13-1 sth1-mIAA7 snf2-mIAA7 before and after 1 h of IAA treatment (final concentration 1 mM). The anti-Pgk1 Western Blot serves as loading control. Data is representative of n = 2 biological replicates. (B) Growth defects of sth1-mIAA7, snf2-mIAA7 and sth1-mIAA7 snf2-mIAA7 strains on IAA plates indicate successful degradation: Five-fold serial dilutions of WT, cdc13-1, cdc13-1 sth1-mIAA7, cdc13-1 snf2-mIAA7 and cdc13-1 sth1-mIAA7 snf2-mIAA7 were spotted on YPD and YPD + IAA (1 mM) and incubated for 48 h at indicated temperatures. Data is representative of n = 2 biological replicates. (C) Linear mixed-effects model of QAOS experiment in Fig 4C for nucleosome evictor mutants: The significance of the difference of increasing ssDNA levels with time after temperature shift to 30 °C was calculated for cdc13-1 sth1-mIAA7, cdc13-1 snf2-mIAA7 and cdc13-1 sth1-mIAA7 snf2-mIAA7 compared to the cdc13-1 control strain. Slope values are provided in S4 Table. (TIFF) [file pone.0352656.s004.tiff]

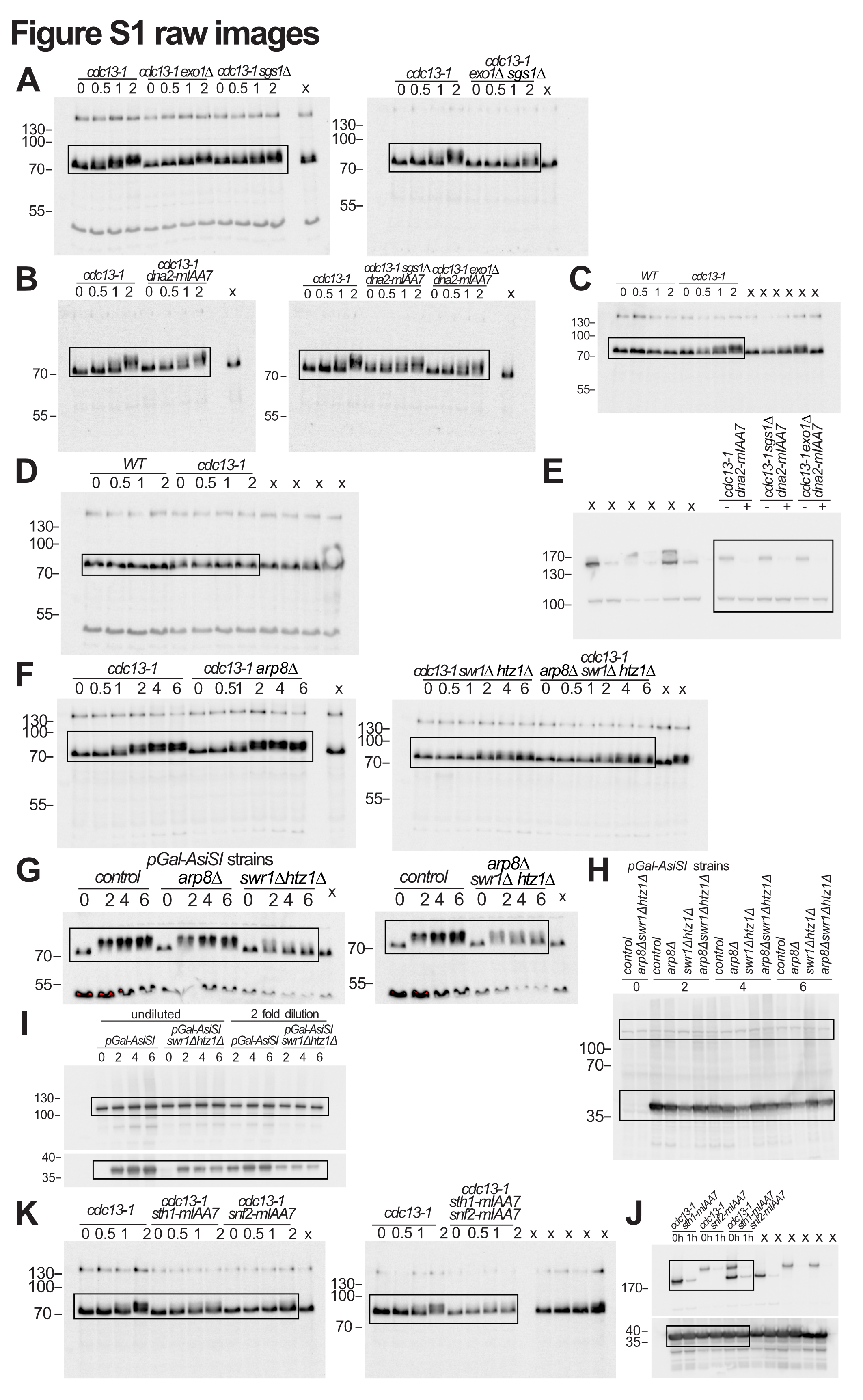

Supplement: S1 Raw Images — (A–K) Uncropped Western Blot images. Rectangles represent the selected areas for the main figures. (A) Anti-Rad53 Western Blots corresponding to Fig 1B. (B) Anti-Rad53 Western Blots corresponding to Fig 1E. (C) Anti-Rad53 Western Blot corresponding to S1A Fig. (D) Anti-Rad53 Western Blot corresponding to S1B Fig. (E) Anti-Flag Western Blot corresponding to S1F Fig. (F) Anti-Rad53 Western Blots corresponding to Fig 2B. (G) Anti-Rad53 Western Blots corresponding to S3A Fig. (H) Anti-HA Western Blot corresponding to S3B Fig. (I) Anti-HA and anti-Cdc48 Western Blots corresponding to S3C Fig. (J) Anti-Rad53 Western Blots corresponding to Fig 4A. (K) Anti-Flag and Anti-Pgk1 Western Blot corresponding to S4A Fig. (TIFF) [file pone.0352656.s005.tiff]
