# Supplementary material for: A role for nucleosome remodellers during resection of deprotected telomeres in yeast
Source: PLoS One. 2026 Jul 10;21(7):e0352656. doi: 10.1371/journal.pone.0352656 (PMC13353985; doi:10.1371/journal.pone.0352656)
Supplement: Attachment — Submitted filename: point-by-point response.pdf [file pone.0352656.s014.pdf]

Point-by-point response:

Response to Reviewer/Editor: We thank the reviewer and reviewing editor for their careful and thoughtful evaluation of our manuscript. We were encouraged by the positive response and that the reviewer finds that our study "provides a useful framework for dissecting chromatin-dependent regulation of resection in a defined telomeric context."

At the same time, we are grateful for the constructive suggestions and thoughtful assessment of the presented experiments and interpretations. We have carefully revised the manuscript to address these points, including new data and analysis, as explained in the point-by-point response below (in blue).

Remarks Reviewer #1:

In this study, Deiser et al. investigated how exonuclease pathways and chromatin context influence DNA double strand break (DSB) repair by homologous recombination following long-range resection. They employ the cdc13-1 temperature-sensitive allele in S. cerevisiae, which induces deprotected telomeres as previously described. Cdc13 is a component of the CST complex, which binds the single-stranded 3' telomeric overhang and prevents its recognition as a DSB. A critical step in homologous recombination (HR) is DNA end resection, which generates a 3' single-stranded overhang that enables strand invasion. To quantify resection, the authors used QAOS (Quantitative Amplification Of Single-stranded DNA), which relies on primers that preferentially amplify single-stranded 3' telomeric DNA. They show that resection requires the exonuclease Exo1, whereas loss of the helicase Sgs1, which part of an alternative resection pathway, does not measurable affect resection, consistent with previous results by Ngo and Lydall (2010). Using an AID-based degron system, they further demonstrate that Dna2, an essential nuclease that typically acts with Sgs1, is also critical for resection. Combined impairment of Exo1 and Dna2 resulted in an almost complete loss of resection. The authors also examine the contribution of the histone variant H2A.Z and the nucleosome remodelers RSC and SWI/SNF to telomere resection. Combined loss of H2A.Z (encoded by HTZ1) and the catalytic subunit of the SwrC complex did not produce detectable changes in resection. This result is different from observations at other genomic sites, where DSBs are induced by an endonuclease and resection is significantly impaired in the htz1 swr1 mutant. In contrast, loss of either Sth1 (RSC) or Snf2 (SWI/SNF) nearly abolished resection in the cdc13-1 system, underscoring the importance of nucleosome remodeling for resection, consistent with other genomic locations.

Overall, the experiments are carefully performed, include sufficient biological controls, and the data are generally convincing and critically discussed. For the most part, the conclusions are supported by the presented results (see major points). Although the cdc13-1 system has previously been used to study DSB repair at deprotected telomeres, this study extends prior work by investigating the roles of Dna2 and the nucleosome remodelers RSC and SWI/SNF, which are difficult to examine due to their essential functions. The authors further propose that the cdc13-1 system can serve as a general model for studying resection, as it appears to recapitulate key features of DNA repair. However, the data also reveal context-dependent differences; for instance, H2A.Z contributes to resection at endonuclease-induced DSBs but does not appear to play a comparable role in resection near telomeres. Moreover, although Dna2 typically functions in conjunction with Sgs1, loss of SGS1 does not measurably affect long-range resection in this system. Therefore, some of the broader conclusions, particularly regarding the general applicability of the cdc13-1 system, should be tempered to reflect these context-dependent differences. Despite these limitations, the study

provides a useful framework for dissecting chromatin-dependent regulation of resection in a defined telomeric context.

Response: We thank the reviewer for the critical evaluation and are pleased they come to the conclusion that our study "provides a useful framework for dissecting chromatin-dependent regulation of resection in a defined telomeric context." We will respond to the comments in detail below.

First, we would like to clarify what may be a misunderstanding in the reviewer's comments. Based on the data shown in Figure 3, we are currently unable to conclude that H2A.Z or the SWR1 complex promotes resection at enzyme-induced DSBs, as DSB induction was very inefficient in the *htz1Δ swr1Δ* double mutant (Fig. 3B). We are not aware of any experiments in the literature that may have overcome this limitation, as such we cannot conclude that H2A.Z or the SWR1 complex would promote resection of enzyme-induced DSBs.

Major

1. It is very difficult to compare QAOS resection data between WT and mutants across separate panels. Plotting WT and mutants within the same graph, while keeping target loci separate, would facilitate direct comparison and improve statistical evaluation.

Response: We thank the reviewer for this helpful suggestion. To facilitate direct comparison between the *cdc13-1* control strain and the respective mutants, we have reorganized the QAOS graphs (Fig. 1D, 1G, 2D, 2E, 4C). Specifically, all QAOS plots are now grouped within a single figure panel, allowing straightforward side-by-side comparison of the different strains while keeping the individual mutant conditions separated. We believe this revised layout substantially improves clarity and makes differences in resection kinetics easier to assess.

In addition, the statistical evaluation of the QAOS experiments is provided in Supplementary Figures S1E, S1H, S1I, S2A, S2B and S4C using linear mixed-effects models, as described in the Methods section. The corresponding graphs offer an alternative for comparison and statistical evaluation of the different mutant strains.

2. I am not convinced that the data support the conclusion that Dna2 acts in conjunction with Sgs1/STR during telomere resection. The authors show that loss of Sgs1 does not affect resection, either alone or in combination with loss of Exo1. This absence of a detectable phenotype contrasts with the reduced resection observed in exo1 mutant and in the dna2 degron allele. Taken together, these results suggest that Sgs1 does not make a substantial contribution to resection in this context, consistent with the previous report by Ngo and Lydall 2010. Accordingly, the conclusion that the sgs1 mutant is epistatic to the dna2 degron strain is not supported by the current data. In the strict sense, epistasis analysis requires that both single mutants display a phenotype, allowing non-additive effects to be assessed. The corresponding statements in the Abstract and Results should therefore be revised to reflect these limitations.

Response: We thank the reviewer for raising this important and justified point. We agree that we cannot claim an epistatic relationship between SGS1 and DNA2, given the absence of detectable phenotypes for the *cdc13-1 sgs1Δ* single mutant. We have now removed all claims of epistasis or functional cooperation from the manuscript and instead focus on the genetic relationship of *EXO1* and *DNA2* gene products. A central result of our study is the observation that the resection defect of *cdc13-1 exo1Δ* is further enhanced by the removal of Dna2 using the degron approach and we therefore conclude that Exo1 and Dna2 mediate independent resection mechanisms after telomere deprotection, similar to what was already known for resection of genomic DSBs. To support this conclusion, we have now added Supplementary Figure S1I, which shows a significant difference for

the *cdc13-1 exo1Δ dna2-mIAA7* mutant compared to the *cdc13-1 exo1Δ* or *cdc13-1 dna2-mIAA7* strain.

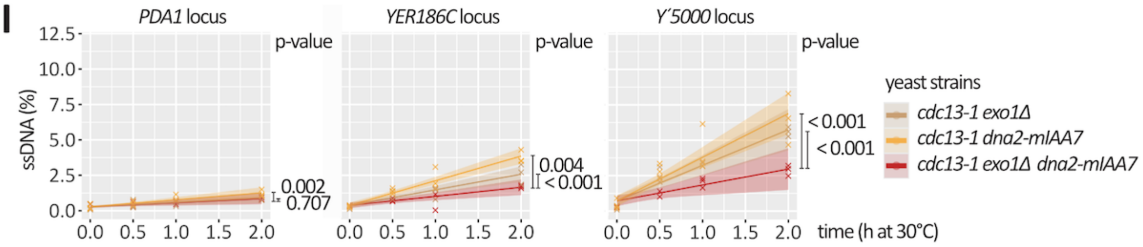

3. While the authors observe reduced resection in the swr1Δ htz1Δ double mutants, they argue that this may be indirect, as ASiSI expression levels are also decreased in this strain background. Although I appreciate this critical assessment, I'm not fully convinced that the moderate change in AsiSI expression based on the western blot data shown in Figure S3B fully explains the nearly complete absence of resection in swr1Δ htz1Δ. Although AsiSI -3HA expression levels vary, the non-specific band used as a loading control also show variability (although signal intensities appear weaker), which complicates interpretation. Without quantitative analysis of western blot signals, including serial dilutions to properly assess quantitative differences in signal intensities, differences in protein levels cannot be reliable assessed. Alternatively, AsiSI expression could be quantified by RT-qPCR, which may provide greater sensitivity. Addressing this limitation would help distinguish whether H2A.Z plays distinct roles at deprotected telomeres and other genomic locations induced by endonucleases.

Response: We thank the reviewer for pointing out that the reduced AsiSI expression may not fully explain the strong reduction in DSB induction. To more quantitatively assess AsiSI expression levels, we followed the reviewer's recommendation and performed quantification of AsiSI-3HA protein levels by Western blot (including analysis of dilution series). These experiments confirm that AsiSI protein levels are reduced to 50 % +/- 30 % in the *swr1Δ htz1Δ* mutant relative to the control strain. The corresponding quantification has been included in Supplementary Figure S3 (S3C and S3D Figs).

Importantly, in our study the primary observation underlying our interpretation for the reduced resection in the *AsiSI swr1Δ htz1Δ* strain is the strongly reduced DSB induction, which we directly measured using Nanopore sequencing (Fig. 3B). This analysis quantifies cutting efficiency at AsiSI sites and demonstrates that DSB delivery itself is strongly impaired in this genetic background. Because efficient resection depends on efficient DSB formation, the reduced resection signal observed in this strain likely reflects this defect in DSB induction.

At the same time, we agree with the reviewer that reduced AsiSI protein levels may not necessarily be the sole explanation for the observed phenotype. For example, altered chromatin organisation has been shown to influence DSB induction (Singh et al., 2021). It is therefore entirely possible that the absence of H2A.Z could also affect accessibility of AsiSI target sites, for instance through increased nucleosome stability or occupancy. We have revised the manuscript to clarify that while reduced AsiSI expression likely contributes to the phenotype, other mechanisms affecting DSB accessibility cannot be excluded.

Changes in manuscript: "We also evaluated DSB induction ("cutting") from the same samples using Nanopore Sequencing, in which we measure reduced coverage of the two nucleotide AT-overhang upon DSB induction (Fig 3B). Notably, we observed similar DSB induction in *WT*, *arp8Δ* and *arp8Δ swr1Δ htz1Δ* mutant strains (Fig 3B). Moreover, resection was comparable among *WT*, *arp8Δ* and *arp8Δ swr1Δ htz1Δ* mutant strains (Fig 3A) indicating that the INO80 complex does not have a major effect on DSB induction or resection. In contrast, we observed strongly reduced DSB-induction in *swr1Δ htz1Δ* cells (Fig 3B). Consistently, we also observed highly reduced DSB resection in the *swr1Δ htz1Δ* strain (Figs 3A-B). Furthermore, Rad53 activation was also reduced in *swr1Δ htz1Δ* cells (S3A

Fig). The reduced DSB induction could be explained by reduced AsiSI expression from the *pGAL1-10* promoter. Therefore, we evaluated AsiSI expression by anti-HA Western Blot (S3B Fig), including dilution series (S3C Fig). Semiquantitative analysis of Western Blots showed reduced AsiSI protein levels in *swr1Δ htz1Δ* cells (50% +/- 30 % S3D Fig), suggesting that changes in AsiSI expression may influence DSB induction. Moreover, H2A.Z and SWR1 could be involved in facilitating DSB induction by promoting DNA accessibility for the AsiSI restriction enzyme by changing chromatin composition or architecture [65].“

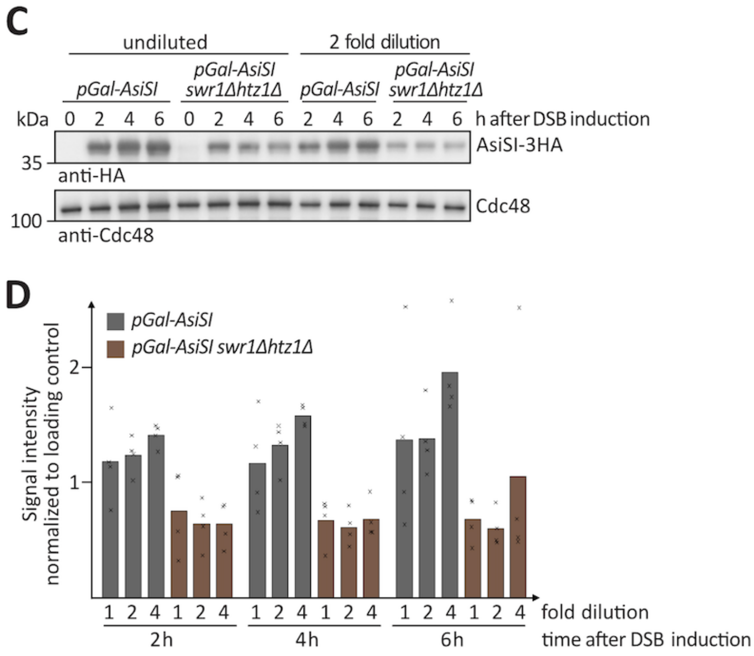

Minor

1. The time points in the prolonged time course experiments (e.g. 2 and 4h) seem to display less ssDNA accumulation than the corresponding 2 h time points in Figure 2E (Y5000). The authors should comment on this discrepancy.

Response: We thank the reviewer for spotting the difference in the resection values of the Y'5000 locus at 2h timepoints between Fig. 2D and Fig. 2E. The experiments were conducted by two different researches and 4 months apart, which has led to a drift/reduction in the experimental values. We are showing the long time-course of Fig. 2E, to ascertain that we cannot detect a resection defect also at the more distal PDA1 locus (29.7 kB away) at late timepoints after telomere deprotection. We now comment on this discrepancy in the Method section.

Changes in manuscript: "Using the same strains, a shift in experimental values of the 2h samples was observed when comparing short and long time course experiments of Fig 2D and Fig 2E, which were conducted with a temporal gap of 4 months and by two different researchers."

2. Did the authors assess whether Fun30 contributes to resection at de-protected telomeres? If not, please clarify the rationale for not testing it.

Response: We thank the reviewer for this comment and share their interest in Fun30 as regulator of resection. Previous work on Fun30 function at enzyme-induced DSBs (Chen et al., 2012, Costelloe et al., 2012, Eapen et al. 2012, Chen et al. 2016, Bantele et al., 2017, Huang et al., 2024) indeed suggests it may be worthwhile testing a role of Fun30 in the *cdc13-1* system as well. The molecular

function of Fun30 is, however, considered more complex, as it counteracts the resection inhibitor Rad9, which acts at *cdc13-1* deprotected telomeres as well. Investigations on the role of Fun30 in the *cdc13-1* system are ongoing and will be reported elsewhere. We have added a brief clarification in the Discussion to better explain the scope of the current study.

Changes in manuscript: "In addition to RSC and SWI/SNF, the nucleosome remodeller Fun30 has also been implicated in regulating long range resection at enzyme-induced DSBs [15,16,24–28]. The molecular mechanism, by which Fun30 promotes resection is still under investigation. Analysis of its role in the *cdc13-1* telomere deprotection system will be reported elsewhere."

3. The authors should explain the rationale why they used the htz1 swr1 double mutant instead of single mutants.

Response: We thank the reviewer for this suggestion. We used the *htz1Δ swr1Δ* double mutant to avoid secondary phenotypes associated with deletion of HTZ1 alone. Previous studies have shown that mutants in the SWR1 complex partially suppress phenotypes arising from H2A.Z loss. This suggests that in the absence of its H2A.Z substrate, the SWR1 complex may remain bound to chromatin and cause additional problems (Halley et al., 2010; Morillo-Huesca et al., 2010). To minimise such indirect effects, we specifically investigated the double mutant combination. We have clarified this rationale in the revised manuscript.

Changes in manuscript: "Furthermore, the *cdc13-1 swr1Δ htz1Δ* strain lacks both the H2A.Z histone variant and the SWR1 complex responsible for its deposition. Using this double mutant avoids secondary phenotypes caused by persistent SWR1 complex activity causing additional defects in the absence of H2A.Z [63,64]"

4. Mentioning of CST and some additional information (histone variant -> H2A.Z, exonuclease -> Exo1, helicase -> Sgs1) in the abstract could improve accessibility for readers less familiar with the topic.

Response: We thank the reviewer for this helpful suggestion. To improve accessibility for readers less familiar with the field, we revised the Abstract to briefly introduce key components of the study. In addition, following the reviewer's general comments, we toned down several statements in the Abstract to better reflect the scope and limitations of our data.

Changes in manuscript: "DNA double-strand break (DSB) repair pathway choice is strongly influenced by DNA end resection, a process in which the 5′ DNA strand is degraded to generate 3′ single-stranded DNA required for homologous recombination. Although the enzymatic mechanisms of resection have been well defined, its regulation by the dynamic chromatin environment surrounding the DNA break remains less clear. Here, we used the budding yeast *cdc13-1* system to analyse DNA end resection after telomere deprotection. Inactivation of Cdc13, a component of the CST (Cdc13–Stn1–Ten1) telomere capping complex, exposes telomeric DNA ends and triggers a DNA damage response. Using this system, we examined the contribution of long-range resection nucleases and chromatin regulators. Analysis of long-range resection nucleases revealed that the Dna2 nuclease contributes to telomeric processing, particularly in the absence of the exonuclease Exo1. Genetic removal of chromatin regulatory factors showed that H2A.Z, a histone H2A variant incorporated by the SWR1 complex, did not significantly affect resection, whereas depletion of the major nucleosome eviction complexes RSC and SWI/SNF impaired resection after telomere deprotection. Together, these results indicate that nucleosome eviction allows for efficient resection at deprotected telomeres and the analogies to long range resection at DSBs illustrate the utility of the *cdc13-1* system for studying long range resection in broader context."

5. Author list: There appears to be a spelling error in the first author's name

Response: Many thanks for spotting this (autocorrect) mistake. It's corrected.

Remarks Reviewing Editor:

Major Issues:

1. With regards to the effects of nucleosome eviction on resection, the authors state that eviction also prevented telomere resection. However, the authors cannot distinguish between resection of the non-nucleosomal telomeric sequences followed by a block in nucleolytic progression and a block in resection at the telomere itself since QAOS does not measure telomeres sequences directly. To resolve this issue, the authors should use native gel electrophoresis of telomeric sequences to look for the influence of mutations in RSC and SWI/SNF complexes on the accumulation of telomeric single-stranded character.

Response: We thank the editor for raising this important point. The QAOS assay used in our study indeed measures accumulation of single-stranded DNA at defined loci located several kilobases away from the chromosome end and therefore reports on resection of sub-telomeric chromatin and beyond, rather than directly measuring resection of the telomere itself. Long-range resection measured by QAOS clearly requires telomere resection, but it does not measure the process directly.

To clarify this point, we revised the manuscript and replaced several instances of "telomere resection" with "resection after telomere deprotection" or similar wording where appropriate. In addition, we added a paragraph to the Discussion explicitly addressing this limitation of the QAOS assay. In this paragraph, we note that it would be interesting in future work to assess whether RSC and SWI/SNF also influence resection within the telomeric repeat tract itself. Direct experimental approaches that measure single-stranded telomeric DNA, such as native in-gel hybridization assays, are technically demanding and were beyond the scope of the present study.

We believe these clarifications address the editor's concern and improve the precision of the manuscript.

Changes in manuscript: (Results): "Resection after telomere deprotection was measured by quantitative amplification of single-stranded DNA (QAOS) at chromosome V, specifically at a sub-telomeric locus (*Y'5000*), as well as at two genomic loci (*YER186C* and *PDA1*). The three tested loci are located at distances 5 kB (*Y'5000*), 14.5 kB (*YER186C*) and 29.7 kB (*PDA1*) from the right end of chromosome V (Fig 1D). We normalised to an internal locus (*PAC2*) and used ssDNA/dsDNA dilution series to obtain relative amounts of resected ssDNA (S1C Fig). By QAOS, we found that after temperature-shift to 30 °C, up to 10 % -15 % of the sub-telomeric *Y'5000* locus became single-stranded, while we did not observe increased ssDNA at 23 °C (Fig 1D and S1D Fig)."

(Discussion): "When interpreting these results, it is important to note that our QAOS assay measures single stranded DNA formation at loci located several kilobases from the chromosome end. It therefore primarily reports on resection after telomere deprotection within adjacent chromatin rather than within the telomeric repeat tract itself. Consequently, our experiments do not directly assess whether nucleosome remodellers influence nucleolytic processing within telomeric DNA. It will therefore be interesting to determine in future work whether the RSC and SWI/SNF complexes also regulate processing of the telomeric repeat tract itself. Given that budding yeast telomeres are largely devoid of canonical nucleosomes, a potential role of these chromatin remodellers in promoting resection of such substrates would point to a function beyond canonical nucleosome eviction."

2. The epistasis data for the influence of sgs1 and eco1 appears to be misinterpreted. Since the sgs1 mutation alone has no influence, its lack of effect in the double mutant provides no meaningful information. This interpretation should be eliminated from the manuscript.

Response: We thank the reviewing editor for emphasizing this critical point, which was also raised by the reviewers. We agree that due to the lack of a significant resection defect of the *cdc13-1 sgs1Δ* mutant, we are unable to assess the epistatic relationship with other mutants. As such we have removed all claims of epistasis or functional cooperation from the manuscript. Instead, we now focus

on the non-epistatic relationship of *exo1Δ* and *dna2-mIAA7* mutants and added Supplementary Figure S1I showing a significant difference for the *cdc13-1 exo1Δ dna2-mIAA7* mutant compared to the *cdc13-1 exo1Δ* or *cdc13-1 dna2-mIAA7* strain. This central result of the paper suggests that resection after telomere deprotection can be mediated by either Exo1 or Dna2 long-range resection nucleases.

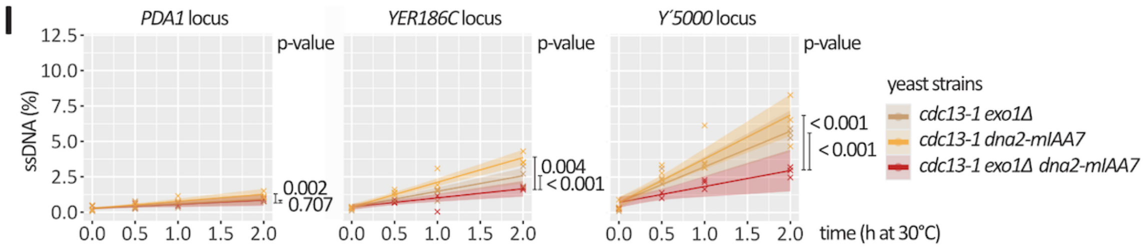

3. Reviewer 1 makes a valid point that the decrease in the Western blot of ASISi expression may be over interpreted. For a better interpretation, a more quantitative assessment is require.

Response: We thank the reviewing editor for this comment. In response to the corresponding concern raised by Reviewer #1, we performed additional quantitative analysis of AsiSI expression by Western blot, including analysis of dilution series. These experiments confirm that AsiSI-3HA protein levels are reduced in the *swr1Δ htz1Δ* mutant compared to the control strain, and the quantification has now been included in Supplementary Figure S3 (S3C and S3D Figs).

Importantly, our interpretation of the resection defect in the *swr1Δ htz1Δ* mutant primarily relies on the direct measurement of DSB induction using Nanopore sequencing (Fig. 3B), which shows that cutting efficiency at AsiSI sites is strongly reduced in the *swr1Δ htz1Δ* background. Because efficient resection depends on efficient DSB formation, the reduced resection signal observed in this strain likely reflects a defect in DSB delivery. At the same time, we acknowledge that reduced AsiSI protein levels may not be the sole explanation for the reduced cutting efficiency. Altered chromatin organization in the absence of H2A.Z could also affect chromatin accessibility to AsiSI-dependent cutting. We have therefore revised the manuscript to acknowledge better this possibility.

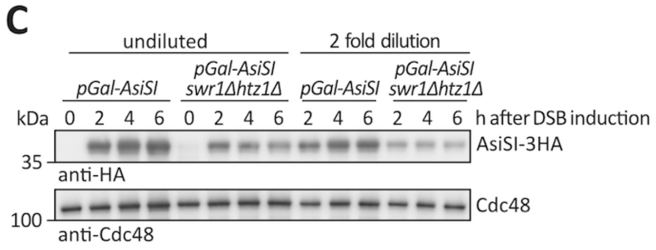

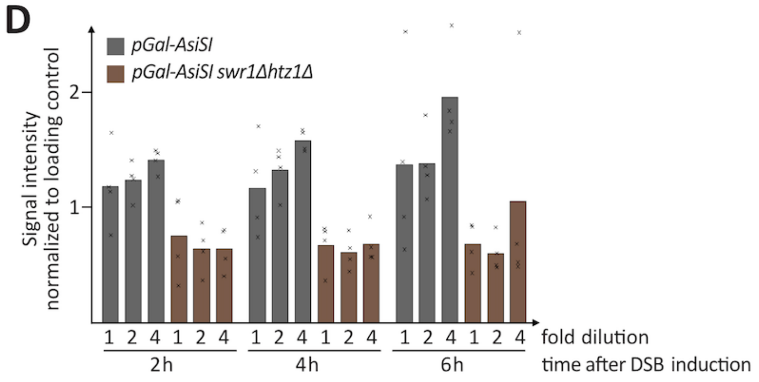

Presentation Issue

4. I agree with the reviewer that the QAOS data would be more easily interpretable if the targets were separated and the wild types and mutants were included in the same graph. I suggest that the authors try this configuration to see if this increases clarity.

Response: We thank the reviewing editor for this helpful suggestion, which is consistent with the comment raised by Reviewer #1. To facilitate direct comparison between the *cdc13-1* control strain and the respective mutants, we have reorganised the QAOS graphs (Figs 1D, 1G, 2D, 2E, 4C). Specifically, all QAOS plots are now grouped within single figure panels, allowing straightforward side-by-side comparison of the different strains while keeping the individual mutant conditions separated. We believe this revised layout substantially improves clarity and makes differences in resection kinetics easier to assess. In addition, the statistical evaluation of the QAOS experiments is provided in Supplementary Figures S1E, S1H, S1I, S2A, S2B and S4C using linear mixed-effects models, as described in the Methods section. These analyses quantify differences in ssDNA accumulation between strains across the time course and provide a complementary representation that facilitates comparison and statistical evaluation of the different mutant strains.
