# Supplementary material for: A role for nucleosome remodellers during resection of deprotected telomeres in yeast
Source: PLoS One. 2026 Jul 10;21(7):e0352656. doi: 10.1371/journal.pone.0352656 (PMC13353985; doi:10.1371/journal.pone.0352656)
Supplement: Peer review history

# Peer review: Decision Letter 0

**DOI:** 10.1371/journal.pone.0352656.r001

**Authors:** Arthur Lustig

8 May 2026

PONE-D-26-13812

A role for nucleosome remodellers during resection of deprotected telomeres in yeast

PLOS One

Dear Dr. Pfander,

Thank you for submitting your manuscript to PLOS ONE. After careful consideration, we feel that it has merit but does not fully meet PLOS ONE’s publication criteria as it currently stands. Therefore, we invite you to submit a revised version of the manuscript that addresses the points raised during the review process.

Please submit your revised manuscript by Jun 22 2026 11:59PM. If you will need more time than this to complete your revisions, please reply to this message or contact the journal office at plosone@plos.org. When you're ready to submit your revision, log on to https://www.editorialmanager.com/pone/ and select the 'Submissions Needing Revision' folder to locate your manuscript file.

Please include the following items when submitting your revised manuscript:

- A letter that responds to each point raised by the academic editor and reviewer(s). You should upload this letter as a separate file labeled 'Response to Reviewers'.
- A marked-up copy of your manuscript that highlights changes made to the original version. You should upload this as a separate file labeled 'Revised Manuscript with Track Changes'.
- An unmarked version of your revised paper without tracked changes. You should upload this as a separate file labeled 'Manuscript'.

If you would like to make changes to your financial disclosure, please include your updated statement in your cover letter. Guidelines for resubmitting your figure files are available below the reviewer comments at the end of this letter.

If applicable, we recommend that you deposit your laboratory protocols in protocols.io to enhance the reproducibility of your results. Protocols.io assigns your protocol its own identifier (DOI) so that it can be cited independently in the future. For instructions see: https://journals.plos.org/plosone/s/submission-guidelines#loc-laboratory-protocols. Additionally, PLOS ONE offers an option for publishing peer-reviewed Lab Protocol articles, which describe protocols hosted on protocols.io. Read more information on sharing protocols at https://plos.org/protocols?utm_medium=editorial-email&utm_source=authorletters&utm_campaign=protocols.

As the corresponding author, your ORCID iD is verified in the submission system and will appear in the published article. PLOS supports the use of ORCID, and we encourage all coauthors to register for an ORCID iD and use it as well. Please encourage your coauthors to verify their ORCID iD within the submission system before final acceptance, as unverified ORCID iDs will not appear in the published article. *Only*  the individual author can complete the verification step; PLOS staff *cannot*  verify ORCID iDs on behalf of authors.

We look forward to receiving your revised manuscript.

Kind regards,

Arthur J. Lustig, PhD

Academic Editor

PLOS One

Journal Requirements:

When submitting your revision, we need you to address these additional requirements.

1.Please ensure that your manuscript meets PLOS ONE's style requirements, including those for file naming. The PLOS ONE style templates can be found at

https://journals.plos.org/plosone/s/file?id=wjVg/PLOSOne_formatting_sample_main_body.pdf and

https://journals.plos.org/plosone/s/file?id=ba62/PLOSOne_formatting_sample_title_authors_affiliations.pdf

2. Please note that PLOS One has specific guidelines on code sharing for submissions in which author-generated code underpins the findings in the manuscript. In these cases, we expect all author-generated code to be made available without restrictions upon publication of the work. Please review our guidelines at https://journals.plos.org/plosone/s/materials-and-software-sharing#loc-sharing-code and ensure that your code is shared in a way that follows best practice and facilitates reproducibility and reuse.

3. Please note that funding information should not appear in any section or other areas of your manuscript. We will only publish funding information present in the Funding Statement section of the online submission form. Please remove any funding-related text from the manuscript.

4. We note that the grant information you provided in the ‘Funding Information’ and ‘Financial Disclosure’ sections do not match.

When you resubmit, please ensure that you provide the correct grant numbers for the awards you received for your study in the ‘Funding Information’ section.

5. Thank you for stating the following financial disclosure:

“Funding by TU Dortmund University”

Please state what role the funders took in the study.  If the funders had no role, please state: "The funders had no role in study design, data collection and analysis, decision to publish, or preparation of the manuscript."

If this statement is not correct you must amend it as needed.

Please include this amended Role of Funder statement in your cover letter; we will change the online submission form on your behalf.

6. When completing the data availability statement of the submission form, you indicated that you will make your data available on acceptance. We strongly recommend all authors decide on a data sharing plan before acceptance, as the process can be lengthy and hold up publication timelines. Please note that, though access restrictions are acceptable now, your entire data will need to be made freely accessible if your manuscript is accepted for publication. This policy applies to all data except where public deposition would breach compliance with the protocol approved by your research ethics board. If you are unable to adhere to our open data policy, please kindly revise your statement to explain your reasoning and we will seek the editor's input on an exemption. Please be assured that, once you have provided your new statement, the assessment of your exemption will not hold up the peer review process.

7. We notice that your supplementary figures are included in the manuscript file. Please remove them and upload them with the file type 'Supporting Information'. Please ensure that each Supporting Information file has a legend listed in the manuscript after the references list.

8. PLOS ONE now requires that authors provide the original uncropped and unadjusted images underlying all blot or gel results reported in a submission’s figures or Supporting Information files. This policy and the journal’s other requirements for blot/gel reporting and figure preparation are described in detail at https://journals.plos.org/plosone/s/figures#loc-blot-and-gel-reporting-requirements and https://journals.plos.org/plosone/s/figures#loc-preparing-figures-from-image-files. When you submit your revised manuscript, please ensure that your figures adhere fully to these guidelines and provide the original underlying images for all blot or gel data reported in your submission. See the following link for instructions on providing the original image data: https://journals.plos.org/plosone/s/figures#loc-original-images-for-blots-and-gels.

In your cover letter, please note whether your blot/gel image data are in Supporting Information or posted at a public data repository, provide the repository URL if relevant, and provide specific details as to which raw blot/gel images, if any, are not available. Email us at plosone@plos.org if you have any questions.

9. If the reviewer comments include a recommendation to cite specific previously published works, please review and evaluate these publications to determine whether they are relevant and should be cited. There is no requirement to cite these works unless the editor has indicated otherwise.

Additional Editor Comments:

In addition to the one external reviewer, the AE also reviewed the paper independently. Those comments are incorporated into the comments below.

Major Issues:

1.    With regards to the effects of nucleosome eviction on resection, the authors state that eviction also prevented telomere resection.  However, the authors cannot distinguish between resection of the non-nucleosomal telomeric sequences followed by a block in nucleolytic  progression and a block in resection at the telomere itself since QAOS does not measure telomeres sequences directly. To resolve this issue, the authors should use native gel electrophoresis of telomeric sequences to look for the influence of mutations in RSC and SWI/SNF complexes on the accumulation of telomeric single-stranded character.

2. The epistasis data for the influence of sgs1 and eco1 appears to be misinterpreted. Since the sgs1 mutation alone has no influence, its lack of effect in the double mutant provides no meaningful information. This interpretation should be eliminated from the manuscript.

3. Reviewer 1 makes a valid point that the decrease in the Western blot  of ASISi expression may be over interpreted. For a better interpretation, a more quantitative assessment is require.

Presentation Issue

4. I agree with the reviewer that the QAOS data would be more easily interpretable if the targets were separated and the wild types and mutants were included in the same graph. I suggest that the authors try this configuration to see if this increases clarity.

[Note: HTML markup is below. Please do not edit.]

Reviewers' comments:

Reviewer's Responses to Questions

**Comments to the Author**

1. Is the manuscript technically sound, and do the data support the conclusions?

The manuscript must describe a technically sound piece of scientific research with data that supports the conclusions. Experiments must have been conducted rigorously, with appropriate controls, replication, and sample sizes. The conclusions must be drawn appropriately based on the data presented.

Reviewer #1: Yes

**********

2. Has the statistical analysis been performed appropriately and rigorously?

Reviewer #1: Yes

**********

3. Have the authors made all data underlying the findings in their manuscript fully available?

The PLOS Data policy requires authors to make all data underlying the findings described in their manuscript fully available without restriction, with rare exception (please refer to the Data Availability Statement in the manuscript PDF file). The data should be provided as part of the manuscript or its supporting information, or deposited to a public repository. For example, in addition to summary statistics, the data points behind means, medians and variance measures should be available. If there are restrictions on publicly sharing data—e.g. participant privacy or use of data from a third party—those must be specified.

Reviewer #1: Yes

**********

4. Is the manuscript presented in an intelligible fashion and written in standard English?

PLOS ONE does not copyedit accepted manuscripts, so the language in submitted articles must be clear, correct, and unambiguous. Any typographical or grammatical errors should be corrected at revision, so please note any specific errors here.

Reviewer #1: Yes

**********

5. Review Comments to the Author

Please use the space provided to explain your answers to the questions above. You may also include additional comments for the author, including concerns about dual publication, research ethics, or publication ethics. (Please upload your review as an attachment if it exceeds 20,000 characters)

Reviewer #1: In this study, Deiser et al. investigated how exonuclease pathways and chromatin context influence DNA double strand break (DSB) repair by homologous recombination following long-range resection. They employ the cdc13-1 temperature-sensitive allele in S. cerevisiae, which induces deprotected telomeres as previously described. Cdc13 is a component of the CST complex, which binds the single-stranded 3' telomeric overhang and prevents its recognition as a DSB. A critical step in homologous recombination (HR) is DNA end resection, which generates a 3' single-stranded overhang that enables strand invasion. To quantify resection, the authors used QAOS (Quantitative Amplification Of Single-stranded DNA), which relies on primers that preferentially amplify single-stranded 3' telomeric DNA. They show that resection requires the exonuclease Exo1, whereas loss of the helicase Sgs1, which part of an alternative resection pathway, does not measurable affect resection, consistent with previous results by Ngo and Lydall (2010). Using an AID-based degron system, they further demonstrate that Dna2, an essential nuclease that typically acts with Sgs1, is also critical for resection. Combined impairment of Exo1 and Dna2 resulted in an almost complete loss of resection. The authors also examine the contribution of the histone variant H2A.Z and the nucleosome remodelers RSC and SWI/SNF to telomere resection. Combined loss of H2A.Z (encoded by HTZ1) and the catalytic subunit of the SwrC complex did not produce detectable changes in resection. This result is different from observations at other genomic sites, where DSBs are induced by an endonuclease and resection is significantly impaired in the htz1 swr1 mutant. In contrast, loss of either Sth1 (RSC) or Snf2 (SWI/SNF) nearly abolished resection in the cdc13-1 system, underscoring the importance of nucleosome remodeling for resection, consistent with other genomic locations.

Overall, the experiments are carefully performed, include sufficient biological controls, and the data are generally convincing and critically discussed. For the most part, the conclusions are supported by the presented results (see major points). Although the cdc13-1 system has previously been used to study DSB repair at deprotected telomeres, this study extends prior work by investigating the roles of Dna2 and the nucleosome remodelers RSC and SWI/SNF, which are difficult to examine due to their essential functions. The authors further propose that the cdc13-1 system can serve as a general model for studying resection, as it appears to recapitulate key features of DNA repair. However, the data also reveal context-dependent differences; for instance, H2A.Z contributes to resection at endonuclease-induced DSBs but does not appear to play a comparable role in resection near telomeres. Moreover, although Dna2 typically functions in conjunction with Sgs1, loss of SGS1 does not measurably affect long-range resection in this system. Therefore, some of the broader conclusions, particularly regarding the general applicability of the cdc13-1 system, should be tempered to reflect these context-dependent differences. Despite these limitations, the study provides a useful framework for dissecting chromatin-dependent regulation of resection in a defined telomeric context.

Major

1. It is very difficult to compare QAOS resection data between WT and mutants across separate panels. Plotting WT and mutants within the same graph, while keeping target loci separate, would facilitate direct comparison and improve statistical evaluation.

2. I am not convinced that the data support the conclusion that Dna2 acts in conjunction with Sgs1/STR during telomere resection. The authors show that loss of Sgs1 does not affect resection, either alone or in combination with loss of Exo1. This absence of a detectable phenotype contrasts with the reduced resection observed in exo1 mutant and in the dna2 degron allele. Taken together, these results suggest that Sgs1 does not make a substantial contribution to resection in this context, consistent with the previous report by Ngo and Lydall 2010. Accordingly, the conclusion that the sgs1 mutant is epistatic to the dna2 degron strain is not supported by the current data. In the strict sense, epistasis analysis requires that both single mutants display a phenotype, allowing non-additive effects to be assessed. The corresponding statements in the Abstract and Results should therefore be revised to reflect these limitations.

3. While the authors observe reduced resection in the swr1∆ htz1∆ double mutants, they argue that this may be indirect, as ASiSI expression levels are also decreased in this strain background. Although I appreciate this critical assessment, I'm not fully convinced that the moderate change in AsiSI expression based on the western blot data shown in Figure S3B fully explains the nearly complete absence of resection in swr1∆ htz1∆. Although AsiSI -3HA expression levels vary, the non-specific band used as a loading control also show variability (although signal intensities appear weaker), which complicates interpretation. Without quantitative analysis of western blot signals, including serial dilutions to properly assess quantitative differences in signal intensities, differences in protein levels cannot be reliable assessed. Alternatively, AsiSI expression could be quantified by RT-qPCR, which may provide greater sensitivity. Addressing this limitation would help distinguish whether H2A.Z plays distinct roles at deprotected telomeres and other genomic locations induced by endonucleases.

Minor

1. The time points in the prolonged time course experiments (e.g. 2 and 4h) seem to display less ssDNA accumulation than the corresponding 2 h time points in Figure 2E (Y5000). The authors should comment on this discrepancy.

2. Did the authors assess whether Fun30 contributes to resection at de-protected telomeres? If not, please clarify the rationale for not testing it.

3. The authors should explain the rationale why they used the htz1 swr1 double mutant instead of single mutants.

4. Mentioning of CST and some additional information (histone variant -> H2A.Z, exonuclease -> Exo1, helicase -> Sgs1) in the abstract could improve accessibility for readers less familiar with the topic.

5. Author list: There appears to be a spelling error in the first author's name

**********

6. PLOS authors have the option to publish the peer review history of their article (what does this mean?). If published, this will include your full peer review and any attached files.

If you choose “no”, your identity will remain anonymous but your review may still be made public.

**Do you want your identity to be public for this peer review?**  For information about this choice, including consent withdrawal, please see our Privacy Policy.

Reviewer #1: No

**********

[NOTE: If reviewer comments were submitted as an attachment file, they will be attached to this email and accessible via the submission site. Please log into your account, locate the manuscript record, and check for the action link "View Attachments". If this link does not appear, there are no attachment files.]

To ensure your figures meet our technical requirements, please review our figure guidelines: https://journals.plos.org/plosone/s/figures

You may also use PLOS’s free figure tool, NAAS, to help you prepare publication quality figures: https://journals.plos.org/plosone/s/figures#loc-tools-for-figure-preparation.

NAAS will assess whether your figures meet our technical requirements by comparing each figure against our figure specifications.

# Author comment: Author response to Decision Letter 1

**DOI:** 10.1371/journal.pone.0352656.r002

4 Jun 2026

We have addressed all concerns by reviewers and editors as indicated. Our response can be found in the file "point-by-point response".

Attachment Submitted filename: point-by-point response.pdf

# Peer review: Decision Letter 1

**DOI:** 10.1371/journal.pone.0352656.r003

**Authors:** Arthur Lustig, Arthur Lustig

15 Jun 2026

A role for nucleosome remodellers during resection of deprotected telomeres in yeast

PONE-D-26-13812R1

Dear Dr. Pfander,

We’re pleased to inform you that your manuscript has been judged scientifically suitable for publication and will be formally accepted for publication once it meets all outstanding technical requirements.

Within one week, you’ll receive an e-mail detailing the required amendments. When these have been addressed, you’ll receive a formal acceptance letter and your manuscript will be scheduled for publication.

An invoice will be generated when your article is formally accepted. Please note, if your institution has a publishing partnership with PLOS and your article meets the relevant criteria, all or part of your publication costs will be covered. Please make sure your user information is up-to-date by logging into Editorial Manager at Editorial Manager® and clicking the ‘Update My Information' link at the top of the page. For questions related to billing, please contact billing support.

If your institution or institutions have a press office, please notify them about your upcoming paper to help maximize its impact. If they’ll be preparing press materials, please inform our press team as soon as possible -- no later than 48 hours after receiving the formal acceptance. Your manuscript will remain under strict press embargo until 2 pm Eastern Time on the date of publication. For more information, please contact onepress@plos.org.

Kind regards,

Arthur J. Lustig, PhD

Academic Editor

PLOS One

Additional Editor Comments (optional):

Reviewers' comments:

Reviewer's Responses to Questions

**Comments to the Author**

1. If the authors have adequately addressed your comments raised in a previous round of review and you feel that this manuscript is now acceptable for publication, you may indicate that here to bypass the “Comments to the Author” section, enter your conflict of interest statement in the “Confidential to Editor” section, and submit your "Accept" recommendation.

Reviewer #1: All comments have been addressed

**********

2. Is the manuscript technically sound, and do the data support the conclusions?

The manuscript must describe a technically sound piece of scientific research with data that supports the conclusions. Experiments must have been conducted rigorously, with appropriate controls, replication, and sample sizes. The conclusions must be drawn appropriately based on the data presented.

Reviewer #1: Yes

**********

3. Has the statistical analysis been performed appropriately and rigorously?

Reviewer #1: Yes

**********

4. Have the authors made all data underlying the findings in their manuscript fully available?

The PLOS Data policy requires authors to make all data underlying the findings described in their manuscript fully available without restriction, with rare exception (please refer to the Data Availability Statement in the manuscript PDF file). The data should be provided as part of the manuscript or its supporting information, or deposited to a public repository. For example, in addition to summary statistics, the data points behind means, medians and variance measures should be available. If there are restrictions on publicly sharing data—e.g. participant privacy or use of data from a third party—those must be specified.

Reviewer #1: Yes

**********

5. Is the manuscript presented in an intelligible fashion and written in standard English?

PLOS ONE does not copyedit accepted manuscripts, so the language in submitted articles must be clear, correct, and unambiguous. Any typographical or grammatical errors should be corrected at revision, so please note any specific errors here.

Reviewer #1: Yes

**********

6. Review Comments to the Author

Please use the space provided to explain your answers to the questions above. You may also include additional comments for the author, including concerns about dual publication, research ethics, or publication ethics. (Please upload your review as an attachment if it exceeds 20,000 characters)

Reviewer #1: For the most part, all of my previous concerns have been addressed. The authors have effectively resolved the points that were previously unclear through improved data presentation, additional experiments, and revisions throughout the manuscript.

While I still believe that presenting the QAOS plots for individual targets and displaying the wild type, single mutants, and double mutants side by side within the same figure would provide a more effective comparison, the revised arrangement adopted by the authors substantially improves the presentation and facilitates comparison of the data.

**********

7. PLOS authors have the option to publish the peer review history of their article (what does this mean?). If published, this will include your full peer review and any attached files.

If you choose “no”, your identity will remain anonymous but your review may still be made public.

**Do you want your identity to be public for this peer review?**  For information about this choice, including consent withdrawal, please see our Privacy Policy.

Reviewer #1: No

**********

# Editor report: Acceptance letter

**DOI:** 10.1371/journal.pone.0352656.r004

**Authors:** Arthur Lustig, Arthur Lustig

PONE-D-26-13812R1

PLOS One

Dear Dr. Pfander,

I'm pleased to inform you that your manuscript has been deemed suitable for publication in PLOS One. Congratulations! Your manuscript is now being handed over to our production team.

At this stage, our production department will prepare your paper for publication. This includes ensuring the following:

* All references, tables, and figures are properly cited

* All relevant supporting information is included in the manuscript submission,

* There are no issues that prevent the paper from being properly typeset

You will receive further instructions from the production team, including instructions on how to review your proof when it is ready. Please keep in mind that we are working through a large volume of accepted articles, so please give us a few days to review your paper and let you know the next and final steps.

Lastly, if your institution or institutions have a press office, please let them know about your upcoming paper now to help maximize its impact. If they'll be preparing press materials, please inform our press team within the next 48 hours. Your manuscript will remain under strict press embargo until 2 pm Eastern Time on the date of publication. For more information, please contact onepress@plos.org.

You will receive an invoice from PLOS for your publication fee after your manuscript has reached the completed accept phase. If you receive an email requesting payment before acceptance or for any other service, this may be a phishing scheme. Learn how to identify phishing emails and protect your accounts at https://explore.plos.org/phishing.

If we can help with anything else, please email us at customercare@plos.org.

Thank you for submitting your work to PLOS ONE and supporting open access.

Kind regards,

PLOS ONE Editorial Office Staff

on behalf of

Dr. Arthur J. Lustig

Academic Editor

PLOS One
